# “One's life becomes even more miserable when we hear all those hurtful words”. A mixed methods systematic review of disrespect and abuse in abortion care

**DOI:** 10.3389/frph.2025.1561707

**Published:** 2025-05-15

**Authors:** Meghana Munnangi, Priya Shreedhar, Regina Gilyan, Hedda Lippus, Dabney P. Evans, Lauren Maxwell

**Affiliations:** ^1^ Hubert Department of Global Health, Rollins School of Public Health, Emory University, Atlanta, GA, United States; ^2^ Department of Obstetrics & Gynecology, UCSF Fresno, Fresno, CA, United States; ^3^ Heidelberg Institute for Global Health, Universitätsklinikum Heidelberg, Heidelberg, Germany; ^4^ Department of Obgyn, Institute of Clinical Medicine, University of Tartu, Tartu, Estonia

**Keywords:** abortion, postabortion care, sexual and reproductive health, women's health, systematic review, pregnant women, adolescent girls, violence

## Abstract

**Background:**

Disrespect and abuse during facility-based abortion and postabortion care (PAC) manifests in various forms, including disrespect, abuse (physical, verbal, and sexual), stigma, discrimination, failure to meet standards of care, neglect, breaches in privacy and confidentiality, misinformation or a lack of information, mistreatment or undignified care, and the presence of protestors. The objective of this mixed methods systematic review was to describe the various forms of disrespect and abuse that women face based on their personal experiences during facility-based abortion care or PAC.

**Methods:**

We conducted a comprehensive literature search in Embase, Medline, and PubMed using Medical Subject Headings (MeSH) and text-based terms targeting disrespect and abuse in facility-based abortion care. The initial search was conducted in 2019, followed by an updated search in 2023. Thirty-eight studies conducted in 20 countries met the inclusion criteria and were included in this review.

**Results:**

The most frequently observed form of disrespect and abuse, identified in 33 studies, was the failure to meet care standards in providing quality abortion care or PAC, particularly in terms of offering adequate and accurate information to women before, during and after the procedures which was identified in 21 studies. Additionally, stigma was reported in 22 studies, disrespect in 19 studies, discrimination in 10 studies, and verbal, physical and sexual abuse, as well as humiliation and condescension, each in 9 studies. The presence and impact of abortion protestors were also looked at in 8 studies.

**Conclusions:**

Our results indicate the need for multi-level strategies to transform healthcare providers' perceptions and attitudes towards women seeking abortion care and other actions at the individual, institutional, and policy levels to provide quality, respectful abortion care and PAC. This systematic measurement of disrespect and abuse in facility-based abortion care and PAC can help understand the distribution of experiences across different groups.

## 1 Introduction

Globally, an estimated 73 million induced abortions occur annually, representing 61% of unintended pregnancies and 29% of all pregnancies (1). Abortion access is hindered by legal, economic, cultural, and structural barriers (2). In legally restrictive settings, pregnant people seeking abortions turn to untrained providers or undergo unsafe abortions that do not conform to minimal medical standards (3). Even in settings where abortion is legally accessible, pregnant people may seek unsafe abortions or illegal abortion services outside of the formal health care system due to stigma, concerns regarding privacy, or fear of disrespect, discrimination or mistreatment from healthcare providers (HCPs) (4–23). HCPs' attitudes, often influenced by personal or institutional biases, can greatly impact abortion experiences, with many providers not fully understanding or accepting the varied reasons for abortion, including socioeconomic concerns, a desire to postpone childbearing, partner-related concerns (e.g., abuse), and risks to personal health (6). Abortion stigma results in, “a negative attribute ascribed to women who seek to terminate a pregnancy that marks them, internally or externally, as inferior to ideals of womanhood” (24). Kumar, Hessini, and Mitchell (24) theorise that abortion stigma exists because of how abortions challenge societal and cultural norms that are placed on women and their roles in motherhood.

### 1.1 Disrespect and abuse during facility-based abortion care

Obstetric violence (OV), a form of gender-based violence (GBV) in reproductive healthcare settings, includes mistreatment, coercion, and neglect that undermine patient autonomy (25, 26). While originally conceptualised around childbirth, OV extends to abortion care, where patients often experience disrespect and abuse from HCPs (27). Bohren et al.'s typology of OV offers a relevant framework for understanding disrespect and abuse in abortion care, including physical, verbal, or sexual abuse, stigma, discrimination, and failure to meet standards of care (neglect, breaches in privacy/confidentiality, misinformation or a lack of information, procedures without consent, mistreatment, or undignified care) (28). Additionally, abortion protestors represent another important source of disrespect and abuse to facility-based abortion care, as external harassment and intimidation at healthcare facilities exacerbate the stigma faced by pregnant persons seeking abortions (29–34). Protestors outside healthcare facilities offering abortions can cause delays in care and increased emotional distress in patients and providers alike (29–34).

Manifestations of disrespect and abuse overlap and are not mutually exclusive (35). Patients' and providers' perceptions of what behaviours constitute disrespect or abuse vary across cultures and contexts (36). Some HCPs may not perceive their behaviours as abusive, particularly if they are learned behaviours or otherwise normalised within healthcare settings (37–40). Paternalistic models of care, characterised by decision-making that disregards patient autonomy and preferences, may further perpetuate disrespect and abuse in abortion care settings (41, 42).

### 1.2 Disrespect and abuse during facility-based postabortion care (PAC)

Postabortion care (PAC) is a critical yet frequently overlooked component of abortion-related healthcare. A recent secondary analysis of data from national service provision surveys across seven low- and middle-income countries shows that there are significant gaps in the provision of basic and comprehensive PAC services (43). The World Health Organization (WHO) describes PAC as tailored medical and supportive interventions, including follow-up visits, management of complications such as incomplete abortion, haemorrhage, infections, anaesthesia complications, and uterine rupture, as well as the provision of contraceptive services (44). However, stigma and provider mistreatment often extend beyond abortion to PAC, resulting in delayed or substandard care (18, 45–48).

### 1.3 Human rights violations and legal frameworks in abortion care and PAC

Disrespect and abuse during abortions and PAC is a violation of human rights. Such abuse violates the 1979 United Nations Convention on the Elimination of All Forms of Discrimination against Women (CEDAW), which requires that state parties must, “eliminate discrimination against women in the field of health care…including those related to family planning” (19). The Beijing Declaration and the Platform for Action, a global agenda for gender equality and empowerment, states that couples and individuals have the right to, “make decisions concerning reproduction free of discrimination, coercion and violence, as expressed in human rights documents” (20). CEDAW further specifies that States must respect, protect, and fulfil human rights related to sexual and reproductive health (SRH) care, including the rights to life, bodily integrity, autonomy, health, and information (19). Human rights bodies have advocated for States to reform laws that criminalise or impede a person's access to safe abortion services (49). Healthcare institutions and providers bear ethical and legal obligations to uphold these rights, particularly in state-funded facilities.

### 1.4 Consequences of disrespect and abuse in facility-based care

Disrespect and abuse can erode trust in healthcare systems and providers, leading to lower healthcare utilisation and poorer health outcomes (50). A study in Tanzania found that women who experienced disrespect and abuse during childbirth were less likely to return to a facility to deliver another child (51). Similarly, a systematic review of studies conducted in Ethiopia revealed that experiences of disrespect and abuse during childbirth influenced women's decisions on where to give birth, often steering them away from choosing institutional deliveries in the future (52). A global systematic review further found that experiencing disrespect and abuse during childbirth was associated with reduced utilisation of maternal postnatal and neonatal care (53). Eliminating disrespect and abuse during abortion care and ensuring high-quality care is an essential step in improving health outcomes for pregnant persons wanting or needing abortions around the world.

### 1.5 Research objective

While the WHO released an official statement aimed at preventing disrespect and abuse during childbirth (54), few studies look at women's experiences of disrespect and abuse during childbirth, and even fewer look at the forms of disrespect and abuse that women face when undergoing abortion care or PAC. To address this gap in the literature, this mixed methods systematic review synthesises qualitative and quantitative evidence from primary studies that explore women's facility-based abortion care and PAC experiences, highlighting the various forms of disrespect and abuse they encounter.

## 2 Methods

This systematic review was conducted in accordance with best practices defined by the Cochrane group (55) and the Preferred Reporting Items for Systematic Reviews and Meta-Analyses (PRISMA) statement guidelines for reporting our findings (56). The systematic review protocol was registered on the PROSPERO International Prospective Register of Systematic Reviews before the searches were conducted (CRD42019124667).

### 2.1 Search strategy

The co-first author MM and reference librarian from Emory University developed a search strategy for Embase, Medline, and PubMed using Medical Subject Headings (MeSH) and text-based terms for disrespect and abuse in facility-based abortion care (See Appendix Tables S1, S2 for the systematic search strategies). The search and subsequent update included articles published between January 1, 1980, and February 19, 2019, and from February 20, 2019, to February 19, 2023. Grounded in Bohren et al.'s typology of OV, we defined “disrespect and abuse” in facility-based care as stigma; disrespect; discrimination; humiliation or condescension; physical, verbal or sexual abuse; failure to meet standards of care, including a lack of information or misinformation, breaches in privacy/confidentiality or a lack of privacy, mistreatment or undignified care, procedures without consent, neglect; and abortion-related protests (28). Grey literature documents were reviewed through Google Scholar using the keywords “disrespect,” “abuse,” and “abortion.” Searches were not limited by study design, publication language, publication type, or geography.

### 2.2 Study screening and data extraction

Included articles met the following criteria: collected primary data related to personal experiences of facility-based abortion care or PAC from women; measured a relevant disrespect and abuse outcome including stigma, disrespect, discrimination, humiliation or condescension, physical, verbal, or sexual abuse, anti-abortion protests on route to or coming from abortion-related care, and failure to meet standards of care which included misinformation or a lack of information, mistreatment or undignified care, breaches in privacy or confidentiality, neglect, and procedures without consent. Data extracted included the study setting, sample population, study design and methods, outcome measures, and information on potential sources of bias.

We conducted deduplication in EndNote and used DistillerSR for the title abstract, full-text screening, and data extraction for the first round of results in 2019. For the first round, two authors (MM, HL) independently conducted the screening and data extraction, except for one Portuguese-language article, which was screened and extracted by one author (LM). Any discrepancies during the title-abstract screening, the full-text screening, or data extraction were resolved by consensus. For the second round of search results in 2023, the co-first author (PS) conducted the title abstract and full-text screening in Covidence and the data extraction directly into the manuscript's tables. The quality of the included qualitative studies was assessed using criteria suggested in a cross-disciplinary expert review of quality criteria for qualitative studies (57) (Appendix Table S3) and using the criteria for quantitative descriptive studies from the Mixed Methods Appraisal Tool (MMAT) version 2018 (Appendix Table S4) (58). Only eight quantitative studies were identified, and as a result, a meta-analysis was not feasible. Ethical approval was not required because the data included in this review contains no identifying information and are publicly available.

## 3 Results

**Figure 1:**
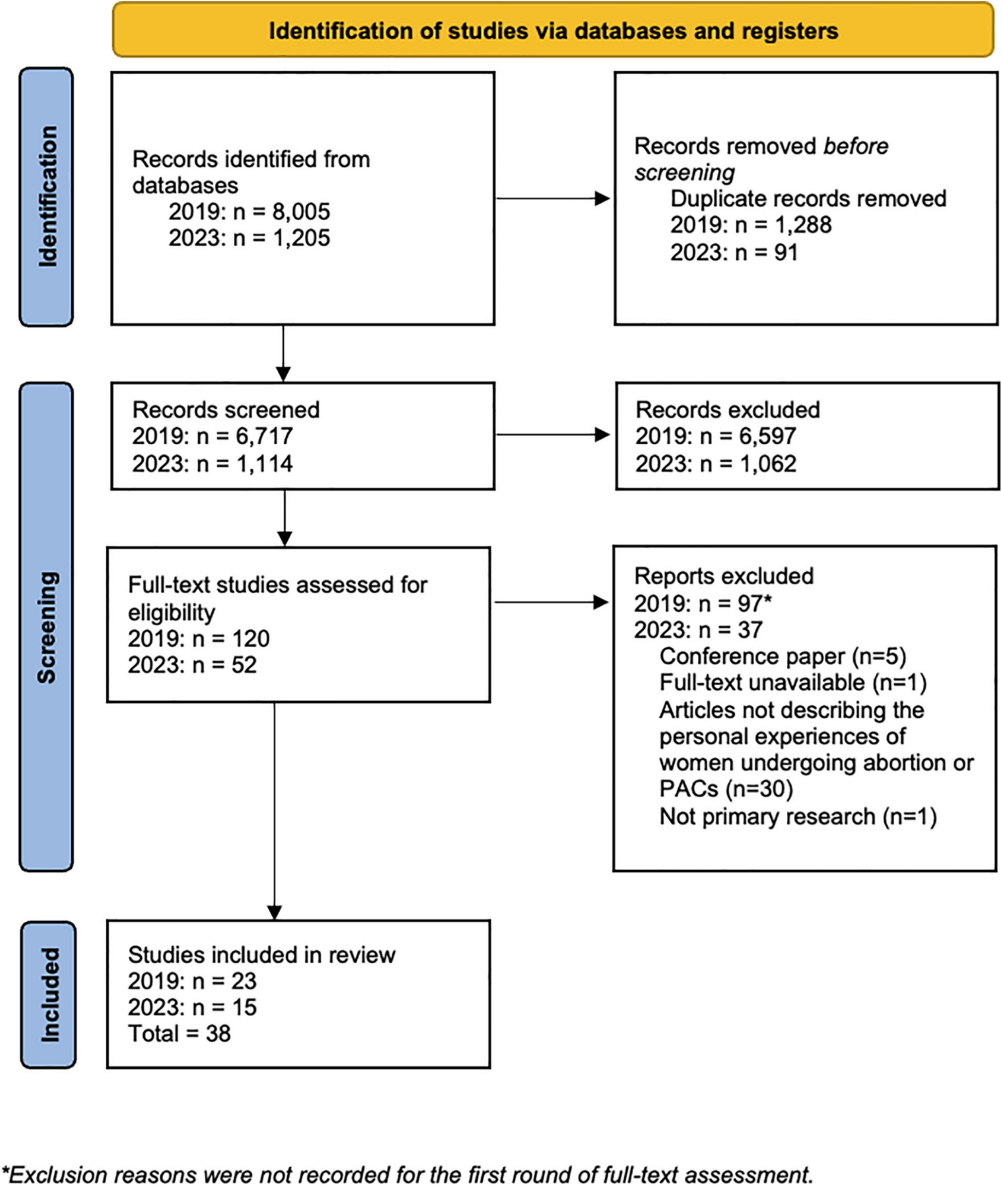
PRISMA flow diagram.

Figure 1 presents the PRISMA flow diagram. Of the 8,005 citations identified during the initial search, we removed 1,288 duplicates and 6,717 titles and abstracts were independently reviewed by two authors. One hundred twenty articles were included in the full-text review, 23 of which met the inclusion criteria. For the updated search, 1,205 citations were identified, of which 91 duplicates were removed, and one author reviewed 1,114 titles and abstracts. Fifty-two articles were included in the full-text review, of which 15 qualified for inclusion. All of the qualitative and quantitative studies included were of moderate or high quality.

We present an overview of the included articles in Table 1. The earliest article was published in 2001 (59). Most articles (*n* = 34; 89%) were published after January 1, 2015. Thirty-seven articles were published in English, and the other was published in Portuguese. Thirty articles used qualitative methods, either in-depth interviews (IDIs; *n* = 30) or focus group discussions (FGDs; *n* = 3), to explore the experiences of individuals receiving abortion care or PAC; eight articles used quantitative surveys (Table 1). Three of the quantitative surveys utilised pre-existing validated scales and questionnaires to look at disrespect and abuse outcomes in abortion care or PAC, including the Discrimination in Medical Settings (DMS) scale (60, 85), the Person-Centered Abortion Care (PCAC) scale (82, 86) and the Quality from the Patient's Perspective (QPP) questionnaire which was modified in the study (84, 87). Other surveys were developed by the paper's authors (29, 30, 67, 76, 81). A detailed overview of each qualitative study's methodology is provided in Appendix Table S3.

**Table 1:** Overview of included studies*.

No	Author, year, area, Geographic region	Sample size, population, study design	Data collection and location	Recruitment strategy	Types of disrespect and abuse faced in relation to facility-based abortion or post-abortion care^†^
1	Aguilar et al., 2023, Northeastern US (60)	163 women who underwent an abortion, Surveys	Self-administered online survey	On the day of abortion, eligible patients were provided a flyer by the recovery room staff that contained a QR code linking to the survey online to be filled out within 1 month of receiving abortion care.	Discrimination, lack of information/misinformation
2	Altshuler et al., 2017, California, US (31)	20 women who underwent an abortion, IDIs	Interviews conducted face-to-face in a non-medical setting or over the phone	Women recruited through Craigslist, flyers at community colleges & public libraries around an area with abortion clinics and birth facilities.	Stigma, breaches in privacy/confidentiality or lack of privacy, impact of protestors, verbal abuse, mistreatment/undignified care
3	Arey, 2023, North Carolina, US (29)	490 women at the abortion clinics, Surveys	Survey administered face-to-face in the waiting rooms of the clinics	Patients and their companions were approached in the outer and back waiting rooms of the abortion clinics by the researcher.	Impact of protestors on women's emotions
4	Baum et al., 2021, Mumbai, India and Eldoret and Thika, Kenya (61)	India: 10 women who underwent an abortion, IDIs; 11 women, FGDs Kenya: 24 women who underwent an abortion, IDIs	Interviews and discussions held in private spaces at the clinics	Women were primarily recruited via health facilities associated with FHOK and FPAI. In Kenya, recruitment also took place through peer educators and other private providers.	Lack of information/misinformation, neglect
5	Becker et al., 2011, Mexico City, Mexico (30)	402 women who underwent an abortion, Surveys	Surveys administered at the health facility after the abortion was complete	Recruitment of women for medication abortion occurred during follow-up visits, while those undergoing surgical abortion were recruited post-procedure.	Lack of information/misinformation, disrespect, breaches in privacy/confidentiality or lack of privacy, impact of protestors
6	Belizan et al., 2020, Tegucigalpa, Honduras (47)	85 women receiving PAC, IDIs and FGDs^‡^	NR	Purposive sampling of women receiving PAC in the hospitals	Refusal of companion, breaches in privacy/confidentiality or lack of privacy, neglect, verbal abuse, lack of information/misinformation
7	Bennett, 2001, Lombok, Indonesia (59)	35 women who underwent an abortion, IDIs 58 women who underwent an abortion, FGDs	NR	NR	Stigma, disrespect, discrimination, lack of information/misinformation, mistreatment/undignified care
8	Bercu et al., 2022, Addis Ababa, Aksum, Mek’ele, Ethiopia (62)	23 women who underwent an abortion, IDIs	Interviews conducted face-to-face at private locations chosen by the participants	Participants were recruited from private clinics and public health facilities depending on the region. Those obtaining abortions at private clinics were contacted for participation through the call centre that originally referred them to a clinic for their abortion. Those obtaining abortions at public health facilities were approached by the local health facility staff prior to their abortion and enrolled immediately after receiving care.	Stigma, disrespect, attempt at dissuasion, lack of information/misinformation
9	Brack et al., 2017, Bogota, Colombia (63)	17 women who underwent an abortion, IDIs	Interviews conducted face-to-face in private consultation rooms at the clinics	Convenience sampling of women by the researcher based on referral by clinic directors, ERC reps and psychologists as well as lawyers from a reproductive rights org.	Stigma, disrespect, verbal abuse, neglect, breaches in privacy/confidentiality or lack of privacy, mistreatment/undignified care, humiliation/condescension
10	Brandi et al., 2018, US (64)	31 women who underwent an abortion, IDIs	Interviews conducted face-to-face in private, non-clinical settings	Convenience sampling of women undergoing abortion at an academic medical centre.	Stigma, lack of information/misinformation
11	Cárdenas et al., 2018, Montevideo, Uruguay (65)	10 women who underwent an abortion, IDIs	Interviews conducted face-to-face; location NR	Convenience sampling of women from a larger quantitative study conducted by the same researchers.	Stigma, disrespect
12	Cano and Foster, 2016, Yukon territory, Canada (66)	16 women who underwent an abortion, IDIs	Interviews conducted by phone or over Skype	A diverse recruitment strategy that included posting study ads on listservs and online platforms, sharing study information through local organisations, and actively using traditional and social media channels.	Disrespect, breaches in privacy/confidentiality or lack of privacy
13	Carroll and White, 2020, Louisiana, US (32)	35 women attending an abortion-related visit, IDIs	Interviews conducted by phone	Clinic staff at the health facilities providing abortions referred women to an on-site research assistant who screened and recruited them for the phone interviews.	Impact of protestors on women's emotions, breaches in privacy/confidentiality or lack of privacy
14	Clyde et al., 2013, Mexico City, Mexico (67)	61 adolescents seeking or have obtained abortion services, Surveys*	Survey conducted as patients left the hospital	Female patients leaving the hospital were approached by the research team for participation in the survey if they accessed abortion services.	Stigma, breaches in privacy/confidentiality or lack of privacy, refusal of companion
15	Deitch, 2019, Democratic Republic of Congo (68)	50 women receiving PAC, IDIs	Interviews conducted in a private room in the health facilities	Purposive selection of participants using PAC registers of 16 healthcare facilities.	Disrespect, breaches in privacy/confidentiality or lack of privacy, neglect, mistreatment/undignified care, lack of information/misinformation
16	Dennis et al., 2015, Massachusetts, US (69)	27 women who underwent an abortion, IDIs	Interviews conducted by phone	Women recruited through flyers posted at local organisations and on Craigslist.	Breaches in privacy/confidentiality or lack of privacy, neglect, impact of protestors
17	DePiñeres et al., 2017, Bogota, Colombia (13)	8 women who were denied an abortion at a private facility (out of which 5 eventually obtained an abortion at the public hospital), IDIs	Initial interviews conducted right after denial of abortion in person at the clinic Second interviews conducted by phone after 2 months of the initial interview	Purposive sampling of women denied abortions at the private clinic.	Stigma, disrespect, discrimination, neglect, verbal abuse, physical abuse, mistreatment/undignified care, humiliation/condescension, breaches in privacy/confidentiality or lack of privacy
18	Doran and Hornibrook, 2016, Rural NSW, Australia (70)	13 women who underwent an abortion, IDIs	Interviews conducted by phone (12 women) and face-to-face in an unspecified location (1 woman)	Women recruited via flyers placed on community notice boards and the backs of public toilet stalls, media announcements, word of mouth, and through women's service.	Stigma, lack of information misinformation, impact of protestors
19	Foster 2020 et al., Canada (33)	305 women who underwent an abortion, IDIs	Interviews conducted by phone or skype	Purposive recruitment based on age and geography through a multi-modal recruitment strategy including outreach efforts through clinics, community organisations, communities at-large, and social media and virtual spaces.	Impact of protestors
20	Heller et al., 2016, Inverness, Scotland (71)	16 women who underwent an abortion, IDIs	Interviews conducted by phone	Clinic nurses contacted women either by phone or face-to-face and informed them of the study and those willing were consented by the nurses for participation and their contact details forwarded to the researcher.	Stigma, neglect
21	Kebede et al., 2018, Addis Ababa, Ethiopia (17)	25 women who underwent an abortion, IDIs	Interviews conducted face-to-face in a location of the woman's choice	Women recruited via the staff at the healthcare facilities.	Stigma, lack of information/misinformation
22	Kilander et al., 2018, Sweden (72)	13 women who underwent an abortion, IDIs	Interviews conducted at a place preferred by the women (their houses, local library) or over skype	Women provided with information on the study by midwives and gynaecologists during abortion counselling. Women who accepted to participate were contacted by the researchers.	Disrespect, lack of information/misinformation
23	LaRoche et al., 2021, Australia (73)	22 women, non-binary and trans men who underwent an abortion, IDIs^§^	Interviews were conducted via telephone or skype	Posting on social media, Australian online classifieds site Gumtree, asking community groups and organisations.	Stigma, confusion regarding legal status of abortion leading to stress during abortion care, lack of information/miscommunication
24	MacFarlane et al., 2017, Istanbul, Turkey (74)	14 women who underwent an abortion, IDIs	Interviewers conducted face-to-face with an interpreter in an unspecified location	Multimodal recruitment strategy that included social media posts, outreach through reproductive health orgs and early participant referrals.	Stigma, disrespect, discrimination, neglect, breaches in privacy/confidentiality or lack of privacy, mistreatment/undignified care, humiliation/condescension, lack of information/miscommunication
25	Madeiro and Rufino, 2017, Teresina, Brazil (75)	78 women with complications after an induced abortion, IDIs	Interviews conducted in a private room within the hospital	Purposive sampling of women admitted for incomplete abortion in the hospital.	Stigma, disrespect, verbal abuse, physical abuse, sexual abuse, neglect, humiliation/condescension, discrimination, mistreatment undignified care, forced sterilisation
26	Makleff et al, 2019, Montevideo, Uruguay (76)	207 women who underwent an abortion, Surveys	Survey administered in-person in a private room at the clinic or by phone	Convenience sampling of women before the third of four mandated visits required for a voluntary abortion; questionnaire administered after the fourth visit upon completion of the abortion.	Stigma
27	Margo et al., 2016, South Carolina, US (77)	45 women seeking abortion services, IDIs	Interviews conducted in private rooms in the abortion clinics	Convenience sampling of women who came in for their abortion at the clinics on the same days as when the interviewer was at the clinics	Stigma, disrespect, neglect, lack of information/misinformation, impact of protestors
28	McCallum et al., 2016, Salvador, Brazil (78)	11 women who underwent an abortion, IDIs*	Interviews conducted in locations suggested by the participants	Purposive sampling of women admitted to the hospital due to spontaneous or induced abortions who were identified from a previous study.	Stigma, disrespect, verbal abuse, neglect, breaches in privacy/confidentiality or lack of privacy, discrimination, lack of information/misinformation, mistreatment/undignified care, humiliation/condescension
29	Mutua et al., 2018, Kenya (18)	21 women receiving PAC, IDIs	Interviews conducted face-to-face at the health facilities	Purposive sampling of patients from 6 health facilities.	Stigma, disrespect, neglect, breaches in privacy/confidentiality or lack of privacy, discrimination, humiliation/condescension mistreatment/undignified care
30	Netshinombelo et al., 2022, KwaZulu-Natal, South Africa (46)	23 women receiving PAC, IDIs	Interviews were conducted face-to-face in a quiet room in the hospitals	Purposive and convenience sampling of women who accessed PACs on the day of their discharge.	Stigma, disrespect, verbal abuse, physical abuse, neglect, humiliation/condescension, mistreatment/undignified care, lack of information/misinformation
31	Otsin et al., 2022, Ashanti, Ghana (79)	24 women who underwent an abortion, IDIs	Interviews were conducted face-to-face (location not specified)	Purposive sampling of women using advertising materials placed on notice boards and other locations within hospitals and providing material on the study to healthcare workers who informed women about the study.	Stigma, neglect, mistreatment/undignified care
32	Ouedraogo and Juma, 2020, Ouagadougou, Burkina Faso (45)	39 women receiving PAC, IDIs	Interviews were conducted face-to-face at various places depending on the participants preferences	Women undergoing PACs identified by observation at different healthcare facilities.	Stigma, verbal abuse, physical abuse, humiliation/condescension, breaches in privacy/confidentiality or lack of privacy
33	Penfold et al., 2018, Western Kenya (21)	22 women who underwent an abortion, IDIs	Interviews conducted face-to-face in a private location convenient to the woman	Women identified in health facilities as part of a larger study and purposely sampled based on the health facility and location i.e., urban or rural.	Lack of information/misinformation
34	Puri et al., 2015, Nepal (80)	12 women who underwent an abortion, IDIs	Interviews conducted face-to-face at a place and time preferred by the women	Women identified in 2 health facilities and purposely sampled based on geographic proximity to the facilities.	Disrespect, discrimination, lack of information misinformation, mistreatment/undignified care
35	Regmi and Madison, 2010, Kathmandu, Nepal (81)	50 women who underwent a second-trimester abortion, Surveys	Surveys administered by researchers or filled out by the women themselves at undisclosed locations	Purposive sampling of women at two hospitals.	Breaches in privacy/confidentiality or lack of privacy, lack of information/misinformation, mistreatment/undignified care
36	Sudhinaraset et al., 2019, Nairobi County, Kenya (82)	371 women who underwent an abortion, Surveys	Survey administered face-to-face in a private space located within the family planning clinics	Purposive sampling of women who received MVA or medication abortion services at the clinics on the same day as recruitment	PCAC score (disrespect)
37	Sunil, 2022, Tamil Nadu, India (83)	16 women who underwent abortion(s), IDIs	Interviews conducted face-to-face at a suitable and comfortable place for the participants	Snowball sampling of married woman who had experienced at least one induced abortion for reasons other than fetal anomaly and sex selection recruited through VHNs.	Stigma, disrespect, verbal abuse, neglect, humiliation/condescension, lack of information/misinformation, discrimination, mistreatment undignified care, forced sterilisation
38	Wallin Lundell et al., 2015, Sweden (84)	708 women who underwent an abortion, Surveys	Baseline survey was completed in the waiting room at the hospital clinic before women underwent an abortion procedure; the 3-month follow up survey was posted to women	All women who requested an induced abortion at one of the 6 public hospitals in Sweden were approached.	Disrespect, discrimination, breaches in privacy/confidentiality or lack of privacy, mistreatment/undignified care, lack of information/misinformation

* Included are only specific details—such as the study population, methodology, recruitment methods, and outcomes—that directly pertain to women sharing their experiences in relation to abortion care or PAC.

^†^ Some outcomes have been placed under broader categories in Table 2.

^‡^ The specific number of women involved in the IDIs and FGDs was not mentioned.

^§^ Out of the 22 people included in the study, 20 identified as women, and two as transgender or gender non-binary.

APS, abortion patient survey; FGD, focus group discussion; FHOK, family health options Kenya; FPAI, family planning association of India; IDI, in depth interview; MS, marie stopes; MVA, medical vacuum aspiration; NGO, nongovernmental organisation; NSW, New South Wales; PAC, postabortion care; PCAC, person-centred abortion scale; VHN, village health nurse.

The most common form of disrespect and abuse, reported in 33 (87%) studies, was failure to meet standards of care for the provision of quality abortion care or PAC, especially in terms of providing adequate and accurate information to women reported in 21 (55%) of studies (Table 2). Stigma was also reported in 22 (58%) studies, disrespect in 19 (50%) studies, discrimination in 10 (26%) studies, humiliation or condescension and verbal, physical or sexual abuse in 9 (24%) studies, and the presence of abortion protestors in 8 (21%) studies.

**Table 2:** Number of studies measuring each type of disrespect and abuse.

Type of disrespect and abuse	Number of studies	Citations
Failure to meet standards of care		
Lack of information/misinformation	21	17, 21, 30, 46, 47, 59–62, 64, 68, 70, 72–74, 77, 78, 80, 81, 83, 84
Mistreatment/undignified care	17	13, 18, 31, 46, 47, 59, 63, 67, 68, 74, 75, 78–81, 83, 84
Breaches in privacy/confidentiality or lack of privacy	16	13, 18, 30–32, 45, 47, 63, 66–69, 74, 78, 81, 84
Neglect	15	13, 18, 46, 47, 61, 63, 68, 69, 71, 74, 75, 77–79, 83
Forced sterilisation	2	75, 83
Stigma	22	13, 17, 18, 31, 45, 46, 59, 62–65, 67, 70, 71, 73–79, 83
Discrimination	10	13, 18, 59, 60, 74, 75, 78, 80, 83, 84
Disrespect	19	13, 18, 30, 46, 59, 62, 63, 65, 66, 68, 72, 74, 75, 77, 78, 80, 82–84
Humiliation/condescension	9	13, 18, 45, 46, 63, 74, 75, 78, 83
Abuse		
Verbal abuse	9	13, 31, 45–47, 63, 75, 78, 83
Physical abuse	4	13, 45, 46, 75
Sexual abuse	1	75
Presence of abortion protestors	8	29–33, 69, 70, 77

### 3.1 Failure to meet standards of care

Failure to meet the expected standards of care, identified in 33 studies, included a lack of information or misinformation, breaches in confidentiality or a lack of privacy, mistreatment or undignified care, neglect, procedures conducted without consent, including forced sterilisation, and contraceptive coercion.

#### 3.1.1 Lack of information/misinformation

Women in 21 studies seeking abortion care or PAC encountered healthcare staff who either refused to provide any information or provided too little or inaccurate information on the procedures conducted, any complications that could arise from the procedures, how to take care of themselves after the procedures and post-abortion counselling including contraceptive counselling (17, 21, 30, 46, 47, 59–62, 64, 68, 70, 72–74, 77, 78, 80, 81, 83, 84). A woman obtaining an abortion in a public hospital in Turkey, despite asking for information on what the HCPs were doing during the procedure, did not receive information and was ignored (74). Another woman in the same study reported very little interaction with the HCPs during her abortion and said, “There was no doctor-patient relationship. I felt like a test subject” (74). In a survey of 402 women who obtained a first-trimester abortion in Mexico City, although the majority felt that sufficient information on the abortion procedure (93%) and PAC at home were provided (87%), around 52% said that HCPs did not talk with them on how they might feel emotionally after the abortion (30). In another survey of 50 women obtaining a second-trimester abortion in Nepal, 42% thought that the communication during their pre-abortion counselling was not clear and informative enough (81). Women undergoing PAC in Honduras and South Africa described feelings that they had not been provided enough information about the procedures and were discharged without adequate information after being prescribed misoprostol or undergoing uterine evacuation (46, 47).

Women seeking abortions in Ethiopia highlighted how they faced misinformation regarding the safety of abortions from HCPs in an attempt to dissuade them from having the abortion (62). Similarly, a woman seeking PAC for an incomplete abortion in South Africa was told by the HCP that she would not have kids after her abortion (46). In Ethiopia, before being able to obtain abortions, women described being denied services by HCPs who had misconceptions about abortion being dangerous. One woman in the study described being told by a doctor, “They told me wherever I go, the abortion will be done using an instrument, and I may even end up dead during the procedure, or I may come out alive. The doctor told me the death is because of severe bleeding” (62). Women also expressed an unmet need for post-abortion contraceptive counselling services, including the benefits, efficacy, and side effects associated with each method and guidance on comparing different contraceptive methods based on the patient's values, needs, and reproductive goals (21, 59, 72, 78). An unmarried woman in Indonesia highlighted how she did not receive information from the doctor who performed her abortion on contraception, the risks of unprotected sex, and that he just said to refrain from premarital sex again (59). Conversely, two separate studies conducted in the US and Sweden found that HCPs had forced women to choose a contraceptive method following their abortion, with one woman specifying that she was coerced into choosing an intrauterine device (IUD) (64, 72).

#### 3.1.2 Breaches in privacy/confidentiality or lack of privacy

Breaches in privacy and confidentiality or a lack of privacy during abortion care and PAC were reported in 16 studies (13, 18, 30–32, 45, 47, 63, 66–69, 74, 78, 81, 84). A few instances of breaches in privacy that women described in the studies included having to show the security guard their identification card to get into the abortion clinic or to identify themselves to the receptionist (31, 78), waiting in line or sitting in waiting rooms with other women (31, 32, 74) including women in labour (78), attending group information sessions at the clinic before their abortion (32), their procedure being conducted visibly in the same operating room as other women (13, 78), and being in a recovery room after the procedure with no privacy and where women (including those in labour) could all see each other (31, 68, 78). In the US, although some women reported that being in communal settings during the abortion and post-abortion process could lend some comfort and support, this also caused physical and emotional discomfort, especially seeing other women going through discomfort, which reinforced a sense of shame and fear of judgement for having an abortion for some women, especially for those living in smaller communities (31, 32). A woman undergoing an abortion in a hospital in Honduras reported that students were standing by the door and watching her procedure and asking the doctor performing her abortion questions (47). In the same study, women reported that reproductive counselling was conducted after the abortion procedure in the presence of other patients (47). In a study conducted in Colombia, a woman requiring an abortion reported being hospitalised for two days without receiving care and her situation becoming a hospital-wide topic of conversation. She reported being scrutinised by the hospital's doctors and nurses, who even ended up calling the police on her (13). In a survey of Nepalese women undergoing second-trimester abortions, 44 out of 50 (88%) said that they were dissatisfied with the level of privacy and confidentiality during their abortion care (81). Around 20% of 61 adolescent girls who obtained an abortion in a hospital in Mexico City indicated that they were not satisfied with their care, and that this was on account of the lack of privacy they experienced (67). In another study of 402 women who obtained a first-trimester abortion in Mexico City, women rated their care and interactions with the staff highly, although around 16% rated the staff as only “somewhat careful” with their personal information (30). This was reflected even in high-income settings, and in a survey of 708 women in Sweden, although around 80% perceived their care as adequate at a 3-month follow-up, almost 15% felt that they did not receive enough privacy and rest during their procedure (84).

#### 3.1.3 Mistreatment/undignified care

Women reported mistreatment and undignified care in 17 studies (13, 18, 31, 46, 47, 59, 63, 67, 68, 74, 75, 78–81, 83, 84). Women receiving abortion services or PAC reported being placed next to women in labour or with women who had given birth in the postpartum ward, which distressed them (13, 46, 68, 78), refusal of pain medications or inadequate pain management (13, 46, 74, 75), lack of anaesthesia at the facility (68) and two or more patients having to share a single stretcher (18). One woman undergoing PAC at a hospital in South Africa described her situation, “I felt horrible pain when the doctor started cleaning my womb. When I told him that I was in pain, he told me, you deserve it” (46). The survey of 50 Nepalese women undergoing second-trimester abortions revealed that 48% did not think that the doctor's commitment to minimising pain with analgesics was adequate (81). Although some women preferred to be awake during the procedure to feel at ease, in control of the situation and to ensure their safety, several women in the US stated that their wishes of being administered general anaesthesia and being unconscious during the procedure were not respected due to medical norms for anaesthesia administration in the US (31).

Women reported being traumatised upon seeing their own or another woman's pregnancy removal and foetus. This occurred either due to the woman being awake during the procedure, lack of a proper set-up in the operating room or ward, or purposely by the HCPs as a way to punish the women for having an abortion or to teach them a lesson (13, 31, 63, 74). A woman undergoing an abortion in the US said of her experience, “They put my baby in a jar that they had just slurped out of me…it was gruesome for me to see” (31). In another study in Colombia, three women receiving inpatient abortion care reported having long-term psychological trauma from nurses presenting the foetus to them in a plastic bag or wrapped in gauze and leaving it at the foot of their bed or in a tub in their hospital room (63). Women also described how they were not asked or given a choice if they wanted the person accompanying them present or not during their abortion care or PAC (47, 67, 74). In one instance in Brazil, a 20-year-old woman experiencing a miscarriage was unable to tell HCPs that she was experiencing a miscarriage rather than an incomplete induced abortion, which influenced the attitudes of the HCPs and the type of care she received. Only once her mother was able to gain access to the hospital and talk to the HCPs did everyone believe she was experiencing a miscarriage (78).

#### 3.1.4 Forced sterilisation

A qualitative study conducted in Southern India reported that sterilisation was suggested to women either as a way to convince them not to abort or as an insult (83). The study also reported a woman's experience wherein she was denied sterilisation after childbirth; however, later on, HCPs at a government hospital would only agree to perform her abortion if her husband permitted her to undergo a sterilisation procedure as well. Another woman in the study was allowed an abortion only if she agreed to a sterilisation procedure as well (83). In another study in Brazil, one participant reported undergoing a non-consensual hysterectomy without prior discussion with the HCP (75).

#### 3.1.5 Neglect

Bohren et al.'s typology of OV describes neglect as abandonment, long delays in receiving care, inattentiveness and other provider behaviours that leave women feeling isolated, ignored, and burdensome during labour and delivery (28). Women mentioned aspects of neglect in abortion care and PAC in 15 studies (13, 18, 46, 47, 61, 63, 68, 69, 71, 74, 75, 77–79, 83). Examples of neglect in care highlighted by women included not receiving a call or check-up from the doctor after the abortion (61, 74). According to a woman who underwent an abortion in India, confirmation that the pregnancy had ended was the most critical indicator of high-quality care (61). Other instances of neglect described by the women were long waiting times or being made to complete administrative work even whilst they were experiencing pain (47, 69, 74, 79), being purposely ignored and being made to wait after having asked for care (13, 18, 46, 63, 74), doctors not acknowledging or treating symptoms such as pain (13, 46, 47, 68), and lack of emotional support from staff (69, 83).

### 3.2 Stigma

More than half of the included studies detailed the experiences of women facing external abortion-related stigma during their abortion care or PAC (13, 17, 18, 31, 45, 46, 59, 62–65, 67, 70, 71, 73–79, 83). Women frequently reported very stigmatising language used by HCPs, including shaming women for the immorality of premarital sex and getting pregnant without being married (18, 59) and judging them for wanting an abortion or PAC (18, 65, 74), including attempting to dissuade them from abortion (13, 31, 46, 62, 63, 73, 83). A woman accessing PAC in South Africa reported being told by a nurse she should not associate herself with other young girls because she would teach them about abortion (46). Nearly a quarter (24%) of 207 surveyed women seeking abortion services at the largest women's hospital in Uruguay reported feeling judged by HCPs during their abortion (76). Other experiences of stigma reported by women in the studies ranged from being passed flyers with anti-abortion content when entering a healthcare facility (13), having to stand in separate lines from others to receive abortion-related information or to enter the hospital for an abortion (67), and having curettage's conducted in an operating theatre in a less visible part of the hospital that is known as the one for “the infected” (78). Studies found that HCPs also often acted as though women were committing a “crime” even when abortion was otherwise accessible through legal means within the country (13, 46, 63, 74, 83).

Restrictive national abortion laws and health facility policies perpetuated a national and/or institutional culture of stigma. One woman in a Colombian study reported that police were called after HCPs accused her of seeking an illegal abortion (13); she then went to another hospital to seek services but still faced stigma as the provider at the second facility stated he would not help her due to his “personal integrity”, and she ultimately obtained an abortion at the facility with another provider (13). During her sonogram, a woman accessing abortion services in another study in Colombia was told by her doctor, “You can already hear the heartbeat; how are you going to kill it?” (63). In a study in Ethiopia, a woman requesting an abortion at a public hospital was told by the HCP, “If you want to abort it, you will sign for it”, implying that the woman had to assume legal responsibility for the abortion (62). In a study in a public hospital in Brazil, 28% of the 78 participants reported that healthcare staff threatened to report them to the police (75). Some women also perceived HCP pressure on them to choose a type of contraception during post-abortion contraceptive counselling to be reflective of the healthcare staff's judgmental attitude towards abortion (64). Women in Burkina Faso obtaining PAC expressed fear of HCPs reactions and stated that they would likely report the women to the police after providing them with treatment (45).

### 3.3 Disrespect

Disrespect during abortion care and PAC was reported by participants in 19 studies (13, 18, 30, 46, 59, 62, 63, 65, 66, 68, 72, 74, 75, 77, 78, 80, 82–84). In the survey of 708 Swedish women who underwent an abortion at the outpatient clinics of public hospitals, almost 23% thought that a deficiency in the provision of abortion care was respectful treatment by healthcare staff (84). In another survey of 353 women undergoing abortions at family planning clinics in Kenya, around 24% of women gave a low score for the Respectful and Supportive Care Sub-Scale of the PCAC scale (82). Across the studies, women have reported facing disrespect from different HCPs, including doctors, nurses, anaesthetists, psychiatrists, administrators, receptionists and security guards. A woman in a study in Colombia reported that a psychiatrist invited a group of students into her hospital room and proceeded to describe her to them as someone with a “severely compromised mental state”, which caused her to have a breakdown and almost leave the hospital (63). Women in the studies in this review have also reported facing disrespect throughout their abortion process or PAC, including during admission to the facility or when interacting with clinic staff or their general practitioner, whilst undergoing the abortion procedure or PAC, during contraceptive counselling and post-abortion counselling, as well as during any post-abortion follow-ups/check-ups. In one study conducted in the US, an 18-year-old Black woman's provider handed her a Planned Parenthood pamphlet and left the room after she mentioned pregnancy termination as an option (77). In another study of women undergoing PAC in South Africa, a woman reported being told by the receptionist, “to go to the next window to get the file because he doesn’t deal with abortion women who are killers” (46). The abortion process in Canada requires multiple contacts with different HCPs, which limits abortion providers' control over women's abortion experiences (66). In one study conducted in Canada, a woman wanting an abortion reported being shown the ultrasound image of the foetus despite requesting the opposite. In the same study, close to half of the participants reported distressing experiences during their ultrasounds from seeing the images, hearing the heartbeat of the foetus or receiving unwanted information about the health of the foetus (66).

### 3.4 Discrimination

Women in 10 studies reported facing discriminatory behaviour from HCPs (13, 18, 59, 60, 74, 75, 78, 80, 83, 84). Study participants contrasted the discrimination they experienced during the abortion or PAC to what they saw as the experience of women at the facility for childbirth (13, 78). A woman in Colombia reported that once the doctors in the maternity hospital realised she was there for an abortion rather than childbirth, they treated her poorly, including delaying her procedure and refusing to give her pain medication (13). In Brazil, a woman recounted her experience of waiting for care due to an incomplete abortion and feeling discriminated against, as even pregnant women who did not require urgent hospitalisation were seen before her (78). Similarly, a woman obtaining an abortion at a private clinic in Nepal, where she had previously been admitted twice for operations and felt treated very well, described the stark difference in the care she received in the abortion ward, where the healthcare staff were rude to her (80). Experiences of discrimination recounted by women in studies conducted in Indonesia, Turkey, Sweden, and Brazil showed that adolescents, unmarried women, women who have had multiple abortions, and women with mental health issues were more likely to experience discrimination during abortion care, including having to pay higher prices for care and receiving a lower quality of care (59, 74, 78, 84). A woman obtaining an abortion in a public hospital in Turkey believed that she would not have had to pay for her abortion at all if she had been married (74). Around 15% of 163 women in the US who obtained abortions at reproductive clinics reported in a survey using the DMS scale that they experienced race- or ethnicity-based discrimination during their abortion (60). The study showed that Black non-Latinx women had the greatest odds of experiencing race- or ethnicity-based discrimination (60). In India, women reported facing discrimination based on their socioeconomic background and caste identity (83). A woman in the study reported, “Then the doctor abused me with my caste identity, remarking, “Your caste people always do this [abortion], and so on”. One's life becomes even more miserable when we hear all those hurtful words” (83).

### 3.5 Humiliation or condescension

Experiences of humiliation or condescension were reported in nine studies (13, 18, 45, 46, 63, 74, 75, 78, 83). In a study in Colombia, a participant was hospitalised for two days without receiving care. During her stay, her abortion became a common topic of discussion among the hospital staff. Nurses would walk by her and stare and ask her why she wanted an abortion (13). A woman in another study conducted in Colombia also reported that she faced condescension from the hospital administrators and HCPs, who feigned ignorance on what an abortion was, and that she was sent all over the hospital in search of someone who could help her (63). Three other studies in South Africa, Turkey, and Kenya found that women who received abortion care or PAC reported that hospital staff or administrators refused to guide them or sent them to different places in the hospitals to try and find someone who could help them (18, 46, 74). In another study in Brazil, a young woman who was admitted to the public hospital for a miscarriage which was mistaken for an abortion was humiliated by the doctor taking care of her who, after performing the curettage, showed her the blood and tissue and said “Oh, see what you have done to your child, everything is putrid inside” (78). Another woman in the study reported feeling humiliated as the doctor reprimanded her for not eating and having a companion with her (78). In a study in India, women reported being humiliated by doctors at public and private healthcare facilities who insulted and scolded them in front of others (83). Women receiving PAC in Burkina Faso also reported feeling shame for being admonished in front of other patients (45).

### 3.6 Verbal, physical, and sexual abuse

Women reported verbal and physical abuse in nine studies (13, 31, 45–47, 63, 75, 78, 83). Verbal abuse included being scolded and yelled at (46, 75, 78, 83), laughed at (13, 47), enduring rude comments and insults (13, 31, 46, 75, 83), being made to feel guilty (13, 45, 46, 63, 83) and told that they were committing a crime or sin by aborting (46, 75), and repeatedly having their decision to abort questioned (63, 83). Women seeking abortion or PAC were labelled as sinners and killers (46, 75) and told that they deserved the pain (46) they experienced during the procedure. In a study in Colombia, a participant reported being in the same room as a woman undergoing a miscarriage at 2 months and a nurse saying to the participant, “Ironic, don’t you see? She wants a baby, and you’re tossing one out” (13). In another study conducted in Colombia, a woman reported being threatened by a nurse that if she continued with the abortion, then the nurse would throw the foetus in the trash. After the abortion, the nurse then proceeded to put the foetus in a plastic bag and whispered to the woman when no one else was around, “I told you that your baby is going to be thrown in the trash” (63). A woman in a study conducted in India reported a senior female doctor at a public hospital telling her, “You would go and lie down to *evannukko* (some man), and is it our job to do abortion for you? … better get operated [female sterilisation]. If we do an abortion, you will go to some other person and become pregnant again” (83). The woman reported feeling ashamed and embarrassed as this happened to her in front of others at the hospital (83). Women in South Africa undergoing PAC experienced verbal abuse from HCPs, including one woman being told that when she died, the baby would be waiting for her in heaven crying. In the same study, another woman reported being told while screaming due to pain, “scream like the time when you were sleeping with your boyfriend” (46). Physical abuse included rough physical examinations from the HCPs and performing abortion-related procedures in a manner that caused women extensive pain (46, 75). In one study in Burkina Faso, some women with an incomplete abortion stated that they preferred misoprostol for evacuation as they perceived that HCPs could hurt them while doing a manual vacuum aspiration (MVA), which was corroborated by the authors' observations that some HCPs used MVAs to make the procedure as painful as possible for women so they could teach them a lesson (45). Women in a study conducted in Brazil reported sexual abuse during abortion care, including HCPs touching their vagina without prior explanation or consent (75). No other studies reported sexual abuse.

### 3.7 Abortion protestors

Eight studies looked at the impact of anti-abortion or anti-choice protestors outside healthcare facilities that provided abortion services (29–33, 69, 70, 77). The studies showed that the presence of protestors created several emotional and logistical barriers to accessing abortion services for women. A study that measured the quality of care at three public sites that offered abortion services in Mexico City reported that 67% of women saw anti-choice protestors outside the facilities. Of these women, 62% were bothered by anti-choice protestors (30). Another survey in the US showed that 397 of 655 (61%) women would have found their experience at the clinics less stressful if there were no protestors (29). Women reported feeling self-conscious, judged, embarrassed, uncomfortable, anxious, threatened and worried that their privacy was being compromised while encountering protestors. The presence of protestors forced some women to choose other clinics that may be further away, and 27% of women visiting abortion clinics in the US in a survey said that the presence of protestors made it dangerous to drive into the clinics (29). Confrontations and difficult interactions with protestors also caused feelings of shame and conflict or exacerbated the feelings of guilt that women were already experiencing. A 15-year-old girl residing in the US and seeking abortion services reported encountering protestors with signs that had pictures of macerated foetuses and that a female protestor approached her and told her that she hoped God would forgive her for murdering her child. This encounter overwhelmed the 15-year-old, who until then had not thought of her 6-week pregnancy as a “child” (31). Another 21-year-old, also in the US, who discovered her pregnancy after ending cancer treatment and an abusive relationship, described feeling very sad after an interaction with a male protestor who said, “You're so beautiful. I'll tell everyone about you in Heaven since you won't be there” (32). A 29-year-old woman who obtained an abortion in Canada reported that “[the protestors] made me feel a little bit more ashamed, but I already sort of felt that way anyways” (33). Women were also angry and frustrated that they had to encounter protestors. In the same study that assessed the impact of protestors on women obtaining an abortion in Canada, participants acknowledged the importance of freedom of thought and expression. Still, they felt that how those rights were exercised was incorrect and unnecessarily traumatic (33). In the study, all participants indicated that although encounters with the protestors were distressing, the protests did not change their decision to terminate the pregnancy (33).

## 4 Discussion

This review synthesised findings on disrespect and abuse in abortion care and PAC from 38 articles in 20 countries. While several systematic reviews summarise the evidence for disrespect and abuse in obstetric care (28, 88–92), to the best of our knowledge, this is the first systematic review of existing evidence on disrespect and abuse in abortion care and PAC. We identified another systematic review and meta-analysis looking at disrespect and abuse during both childbirth and abortion. However, the review was limited to quantitative studies of disrespect and abuse in obstetric and abortion care in Latin America and focused on estimating the prevalence of any form of disrespect or abuse rather than describing different types of disrespect and abuse (93).

The most common forms of disrespect and abuse identified in this review were failure to meet standards of care (33 studies), followed by stigma (22 studies), disrespect (19 studies), discrimination (10 studies), humiliation/condescension (9 studies), verbal, physical and sexual abuse (9 studies), and the presence of abortion protestors (8 studies). The Global Doctors for Choice Network has developed a conceptual framework in their report, “Obstetric Violence and Abortion. Contributions to the Debate in Colombia”, which identifies the factors that underpin disrespect and abuse during abortion care, including at the individual, institutional, community/societal, and governmental/legal levels (25). We use this framework to summarise our findings and formulate recommendations to reduce the disrespect and abuse that women face when accessing facility-based abortion care or PAC.

### 4.1 Individual and societal factors

Attitudes and opinions at the individual and societal levels shape disrespect and abuse in abortion care and PAC. High-quality abortion care involves respectful providers that protect and uphold patient's rights, privacy, and decision-making processes without judgement (94). Women in the 38 studies included in this review reported not being provided with high-quality abortion care and PAC due to disrespectful and abusive behaviours from their HCPs. These behaviours are shaped by institutional norms and procedures and societal and cultural taboos, including the stigma surrounding premarital sex and abortion (18, 59, 74). Studies identified discriminatory attitudes and practices against younger and unmarried women obtaining abortions or PAC, reflecting deep-rooted societal and cultural views on premarital sex (59, 74, 78). Women also noted that the personal attitudes of HCPs toward abortions, influenced by social, cultural and religious views, led them to ignore women who were trying to seek services, disrespect and shame them, and sometimes try to dissuade them from going through with the termination, often by providing false or misleading information on procedure-related risks (13, 18, 46, 62, 78, 83). HCPs' views that the women were morally wrong in their decision to abort also led to failures to uphold standards of care, including denying the women painkillers or lack of pain management (13, 46, 74, 75), providing treatment only conditionally, i.e., if the woman also agreed to sterilisation (83), lack of emotional support (69, 74, 83), and using stigmatising or abusive language. Studies found that protestors outside abortion clinics made women feel judged, threatened, and worried that their privacy was being compromised (29–33, 69, 70, 77). Addressing disrespect and abuse in abortion care must also include managing external harassment and intimidation from protestors, as this further exacerbates stigma and undermines pregnant people's rights to respectful abortion-related healthcare.

Marginalised populations have both a more challenging time accessing SRH services and a heightened risk of experiencing disrespect and abuse during their care (95). Racial, ethnic, and caste-based discrimination were highlighted by women in the studies included in this review (60, 83). We also found that younger, unmarried women, women with mental health issues, and women undergoing a repeat abortion could be at a higher risk of experiencing disrespect and abuse in abortion care (59, 74, 76, 78, 84).

### 4.2 Institutional factors

Failure to meet standards of care for the provision of quality abortion care or PAC was reported by women in 87% of studies included in this review, highlighting the important role of institutional factors. These factors include availability of dedicated spaces for service provision, clear and supportive institutional guidelines and policies, and an adequate number of sufficiently trained HCPs (44, 96, 97). Studies in this review reported on women's experiences of lack of spaces and processes dedicated to abortion care and PAC resulting in perceived compromised privacy and confidentiality for those seeking these services (13, 31, 46, 47, 68, 78). Institutional policies and standards of care can inadvertently stigmatise patients by integrating abortion care within maternity wards or general obstetric services, highlighting the need for careful policy design and service implementation that prioritises patient dignity and privacy (97). Because of the societal stigma that surrounds abortion, women go to great lengths to maintain the privacy of their abortion in both legally restrictive and permissive settings (16, 98). Healthcare facilities should ensure their policies and staff maintain the privacy and confidentiality of all patients.

Paternalistic health care was a common theme, and doctors and nurses often undermined women's decision-making, questioned their morals and values, did not provide adequate information to women including sufficient contraceptive counselling (21, 59, 72, 78), uphold standards of care for pain management (13, 46, 74, 75), respect women's choice to have a companion present (47, 67, 74), and neglected women including making them wait for a long time (47, 69, 74, 79). Patient-centred care, including abortion care and PAC, should focus on upholding a patient's fundamental human rights, including their rights to bodily autonomy, non-discrimination, highest quality care, and privacy (99). Women undergoing abortion care have the right to be affirmed as moral decision-makers, determine their involvement in their care, and receive care that is provided discreetly and without judgment (31). To provide quality abortion care, institutions must respect a patient's right to legal abortion and implement policies and education programs to train and support healthcare staff to provide safe and ethical comprehensive abortion care.

High-quality abortion care requires that HCPs are trained in education, counselling, informed consent, skilled clinical assessment, pain and side effect management, identification and management of serious complications, as well as contraceptive counselling and provision (49). Quality abortion care includes accurate and clear contraceptive education and counselling to women in healthcare facilities (100). A systematic review of the attitudes and behaviours of maternal HCPs found that the negative attitudes towards women seeking abortions held by physicians in low- and middle-income countries required long-term investments in infrastructure, education, and communication skills to prevent disrespectful and abusive behaviours towards abortion patients in the next generation of HCPs (101). Educating and training providers on patient-centred abortion care and PAC is essential to improving the quality of care in healthcare institutions. Abortion values clarification and attitude transformation (VCAT) workshops should be held with community leaders, religious leaders, policymakers, and HCPs worldwide to shift stigmatising attitudes and behaviours (102).

Studies included in this review found that HCPs pushed women seeking abortions to undergo sterilisation or coerced women to choose contraception (64, 72, 83). Developing evidence- and rights-based post-abortion counselling would improve the quality of abortion care and address participants' calls for more social support during abortion care. Quality post-abortion contraceptive counselling must be voluntary and should always incorporate the patient's values and needs. The provision of contraception is a critical component of quality abortion care. Healthcare professionals should provide clear, unbiased information about all available contraceptive options, regardless of factors including abortion history, race, ethnicity, marital status, and age, while fully respecting an individual's decision, including their right to decline contraception (100, 103, 104). Forced sterilization is a human rights violation involving removal of a person's ability to reproduce through coercion or without obtaining their informed consent (105), and it disproportionately affects marginalized populations (106, 107). Preventing this abuse requires clear communication, rigorous informed consent processes, comprehensive human-rights-based training for HCPs, and robust oversight and accountability mechanisms within healthcare systems to protect reproductive autonomy (108, 109).

### 4.3 Legal factors

Continuing an unwanted pregnancy can be emotionally taxing and may prolong women's contact with violent partners (110). Legal, financial, and geographical barriers to accessing abortion care and PAC can be detrimental to a woman's emotional and physical health, especially if they are denied access to an abortion (71, 79). Legal access to abortion varied across the 20 countries included in this review. We summarise the legal and social context for these countries in Table 3. In legally restrictive environments, abortions occur as frequently as they do in countries with fewer or no legal restrictions on abortion access (135). Several countries where studies were conducted ban abortion outright or only permit abortions under certain conditions (Table 3). Findings from this review are in keeping with other studies that suggest that legal restrictions push women to seek illegal abortions outside of the health system. International health organisations, like the WHO, have introduced guidelines for countries to reduce barriers to safe abortion services (44), but societal values that condemn abortion persist. Before the overturning of Roe vs. Wade (133, 134), the US, which was considered to have a less restrictive abortion law at the national level, had enacted measures that effectively prevent or restrict access to abortions at the state level, like targeted regulation of abortion providers (TRAP) laws and foetal heartbeat bills (136, 137). Studies in this review have shown that in settings with anti-abortion national laws and restrictive policies, the stigma around abortion is perpetuated, increasing the barriers to accessing abortion care and PAC, as well as fostering disrespectful and abusive behaviours from HCPs (13, 62, 68, 79, 83). In such legally restrictive environments, abortion providers themselves may experience stigma, legal and ethical dilemmas, professional risks, isolation, burnout, and increased emotional distress, all of which can strain the patient-provider relationship (97, 138, 139). However, despite these challenges, some HCPs have actively advocated for abortion access even at personal and professional risk. This includes actions such as utilising telemedicine across restrictive jurisdictions (140, 141) and publicly advocating against restrictive laws (142). A study included in this review that also interviewed HCPs providing abortion care and PAC found that, despite the difficulties, many see their work as essential to saving lives and providing support to women in need, which emphasises a strong philosophical commitment among HCPs to prioritise care (45).

**Table 3:** Legal and policy context across the countries in included studies.

Country	Legal and policy context
Australia	Abortion is legal in all states; however, the gestational limits vary between states (beyond which restrictions apply, another doctor's opinion must be sought, etc.). Providers in most states are allowed conscientious objection and negative attitudes among them toward women obtaining abortions are common. Women in rural areas may face more barriers to access quality, abortion care (70, 111).
Brazil	Abortions are legally allowed only when the pregnant woman's life is at risk, the pregnancy is a result of rape, or in the case of fetal anencephaly. Abortion is still a common event in women's lives in Brazil, with a national 2021 survey reporting that 10% of women have had an abortion. Many women undergo unsafe abortions and access healthcare services for PAC or complications. The Brazilian Supreme Court is considering a case that will decriminalise abortion up to 12 weeks of gestation (75, 78, 112, 113).
Burkina Faso	Abortions are legally allowed only in instances of rape, incest, fetal impairment, or when necessary to preserve a woman's life. Abortions for reasons other than these can result in imprisonment and heavy fines. The abortion rate is 25 abortions for every 1,000 women aged 15–49. Knowledge of the condition-based legal status of abortion is low and most abortions take place under secrecy and with a lot of fear and stigma. Estimates are that around 43% of abortions resulted in complications (45, 114).
Canada	No legal restrictions on abortion. Safe access zones and prohibition of protest activity within a defined distance from healthcare facilities, pharmacies, and GP offices are in place (115).
Colombia	In 2006, Colombia's government overturned a complete ban on abortion and decriminalised abortions for rape, incest, fetal anomaly, or woman's life at risk. Primary health facilities could provide abortions up to 15 weeks, after 15 weeks they needed to be performed at a higher-level facility. Providers were allowed conscientious objection, and, in practice, many women were denied legal abortion services or faced numerous barriers to accessing abortion care (13, 63). In February 2022, the Colombian Courts repealed the existing criminal law on abortion and legalised it up to 24 weeks, under the same conditions as the 2006 ruling (116).
Democratic Republic of the Congo	Abortion is prohibited in the Democratic Republic of Congo (DRC), resulting in restricted access to safe services. Recent signing, ratification, and publication of the Maputo Protocol in the DRC official journal suggests a potential move towards inclusion in the national law, but the implementation process is anticipated to be gradual. Consequently, women continue seeking abortions covertly, often under precarious conditions, and the availability of post-abortion care, as mandated by the national health policy, faces challenges, further impacting women's reproductive health (68, 117).
Ethiopia	In 2005, abortion was expanded to be legal in cases of rape, incest, fetal impairment, danger to the pregnant woman's life, if the woman is physically/mentally unprepared for childbirth or if she has disabilities or is a minor. Despite the liberal law for abortion, a significant number of abortions occur outside of healthcare centres and require PAC for complications. A recent study showed that only 62.4% of self-reported abortions were classified as safe (118, 119).
Ghana	Ghanian criminal code states that abortion is permitted only in cases of rape, incest, fetal abnormality, or woman's life is at risk. The Ghanaian Ministry of Health has developed abortion-related protocols and guidelines targeted at young persons and other groups at risk. However, women are often not aware of the law and abortion is stigmatised leading to around 70% of abortions being self-induced and unsafe (5, 120, 121).
Honduras	Abortion is completely banned under any circumstances in the country's constitution. It is close to impossible to access safe abortion services. PACs are of poor quality (47, 122).
India	Abortion is legal until 20 weeks’ gestation for all women and up to 24 weeks’ gestation under special circumstances such as rape, incest, being a minor, change in marital status, disabilities, fetal anomalies and those living through disasters or humanitarian crises. Stigma, poor quality of abortion care and PAC, and many other barriers still exist (83, 123, 124).
Indonesia	Abortion is illegal in Indonesia unless the woman's life is at risk, severe fetal anomalies, or rape. In July 2024, regulations were changed to allow abortions in these circumstances from 6 weeks till 14 weeks gestation (125). However, abortions are common, with the rate being 25 abortions per 1,000 Indonesian women of reproductive age. Around 79% of these are estimated to be unsafe. Legally, only obstetricians, gynaecologists, or doctors with special training can do abortions, however, a variety of healthcare providers (nurses, midwives, TBAs) provide illegal, unsafe abortions. Abortions and premarital sex are highly stigmatized (59, 126).
Kenya	Abortion is illegal unless in the opinion of a trained medical professional there is a need for emergency treatment or the pregnant woman's life is at risk or if permitted by another law. Due to the highly restricted abortion laws in Kenya, many women undergo unsafe abortions and require PAC for an incomplete abortion (18, 21, 127).
Mexico	In September 2023, the Supreme Court of Mexico decriminalised abortion on a federal level. Prior to this, the legalisation of abortion varied at a state level, with Mexico City being the first state to legalise abortion on request up to 12 weeks of gestation in 2007. Work still needs to be done to address the social stigma around abortion in the country (30, 67, 128).
Nepal	Abortions were conditionally legalised in 2002, and thereafter the legal criteria were revised in 2018 with the Safe Motherhood and Reproductive Health Rights Act. The act permits abortion up to 12 weeks of pregnancy on request and up to 28 weeks under the conditions that the pregnancy is a result of rape, incest, the woman has HIV or other incurable diseases, or if the woman has certain mental health conditions. Abortions are also allowed if the pregnancy poses a danger to the woman's physical or mental health or the presence of foetal abnormalities; in these cases, a doctor's recommendation is required. The Nepali government has implemented policies to improve access to safe and legal abortion however, many women still face barriers, including stigma. Services are still largely inaccessible for low-income, marginalised, and geographically isolated women (80, 81, 129).
Scotland	The United Kingdom allows abortions during the first 24 weeks of pregnancy provided that two doctors agree that an abortion will cause less harm to a woman's physical or mental health or that of her children than continuing the pregnancy (130). In October 2024, a new law banning protests, including silent vigils and prayers, within 150 meters of clinics with abortion services came into effect (131).
South Africa	Abortions allowed up to 12 weeks with no reason required; for socioeconomic, rape, incest, and medical reasons up to 20 weeks; and only if the woman or fetus’ life is in danger or serious birth defects are determined for an abortion above 20 weeks. For abortions above 20 weeks, two medical professionals must approve. Abortion is highly stigmatised and there is a lack of willing providers, treatment delays and mistreatment. Despite legalisation of abortion, women are still undergoing illegal and unsafe abortions because of perceived poor care and stigma (14, 15).
Sweden	Abortion is legal for any reason up to 18 weeks of pregnancy and thereafter for severe indications of medical risk. Post-abortion contraceptive counselling is mandatory. Medical abortions are increasingly being performed at home and midwives can now perform medication abortion care (72, 84).
Turkey	Abortions are allowed up to the 10th week for any reason and up to the 20th week for foetal anomalies, rape, incest, or danger to a mother's health. Married women require spousal consent, minors require parental consent. Abortion is highly stigmatised and access to medication abortion is limited (74, 132).
United States	In June 2022, the Supreme Court overturned previous court rulings regarding abortion rights, including the Roe vs Wade decision of 1973 which effectively legalised abortion across the United States until the point of foetal viability (24 weeks) and prevented states from imposing overly restrictive regulations on a woman's ability to obtain an abortion during all three trimesters of pregnancy. At present, given the absence of a constitutionally safeguarded right to abortion care, states have the liberty to establish stringent legal boundaries concerning the accessibility of abortion, many of which have already done so (133, 134).
Uruguay	Uruguay decriminalised abortion before 12 weeks in 2012. A woman requesting an abortion must attend 4 visits: an ultrasound/lab tests and confirmation of abortion decision (1st visit), counselling session with a committee of professionals (2nd visit), abortion procedure (3rd visit), and follow-up/contraceptive counselling (4th visit). The women must also undergo a mandatory 5-day reflection period between the 2nd and 3rd visits. Healthcare providers are allowed conscientious objection to performing the abortion and women have reported feeling judged by healthcare providers during the abortion process (76).

DRC, democratic Republic of Congo; GP, general practitioner; HIV, human immunodeficiency virus; PAC, postabortion care; TBA, traditional birth attendant.

Although the WHO has recognised disrespect and abuse in obstetric care as an essential issue and encouraged countries to implement laws and policies that address disrespect and abuse in obstetric care (54), most countries do not have policies to prevent violence during abortion care. There is little to no legal recourse or compensation for victims of disrespect and abuse and no or limited accountability for HCPs that violate their patient's human rights during abortion care and PAC. More countries need to propose and enforce laws that reject any form of violence against women, including disrespect and abuse in SRH services. National and local laws, institutional policies, and the codes of conduct for professional associations must uphold pregnant people's fundamental rights to access safe, quality healthcare. They must hold HCPs accountable for disrespectful and abusive treatment of pregnant people seeking abortions and PAC. Furthermore, increasing access to safe medication abortion in early pregnancy, particularly self-managed abortion with medications such as misoprostol and mifepristone, may also serve as an effective strategy to protect pregnant people from experiencing disrespectful or abusive care in healthcare facilities (143). Self-managed medication abortion provides an important alternative pathway for pregnant people seeking abortion services in legally restrictive or healthcare environments where there are increasing barriers to accessing abortions (144).

### 4.4 Strengths, limitations, and recommendations for future research

This review provides a comprehensive summary of the published evidence from both qualitative and quantitative studies of disrespect and abuse in abortion care and PAC, regardless of language or geographic location. To the best of our knowledge, this is the first systematic review that identifies a range of disrespect and abuse commonly encountered in abortion care and PAC, varying from failure to uphold standards of care to stigma within and outside of the health facility and verbal and physical abuse. These issues are prevalent at multiple care levels, highlighting systemic challenges in the provision of respectful, quality abortion care and PAC.

One limitation of this review is the overrepresentation of studies from the Americas, particularly the US. This imbalance underscores the necessity for more research in other regions, including Europe, Asia, and Africa, to understand the scope of disrespect and abuse in abortion care and PAC and how women's experiences differ across cultures and legal settings. Categories of disrespect and abuse overlap, and similar behaviours may have been described and categorised differently across studies by the authors’ understanding of the categorisation of disrespect and abuse outcomes.

Another limitation of this review is that certain experiences of disrespect and abuse in abortion care and PAC by women may reflect standard healthcare practices rather than intentional mistreatment by HCPs. Routine aspects of care, such as shared waiting areas, hospital registration processes, and post-operative spaces, as well as pain medication protocols, may be perceived as mistreatment or breaches in privacy, especially when communication is lacking. Additionally, institutional constraints, such as limited resources or staffing shortages, may shape abortion care and PAC delivery in ways that patients experience as disrespectful. While these factors provide context, they do not justify the persistence of negative patient experiences (28). The integration of abortion care into maternity or general obstetric services may also inadvertently contribute to stigma, emphasizing the need for policies that prioritise the dignity and privacy of those seeking abortion care or PAC.

Included studies were affected by recall bias, social desirability bias, and sampling bias. Multiple articles interviewed women within 5–15 years after their abortion, which may have impacted the accuracy of their reports (31, 66, 70, 73, 74). Several studies also conducted their interviews or questionnaires onsite at the healthcare facilities (18, 46, 47, 61, 63, 68, 75, 77), which may have led to social desirability bias given that participants may not have felt comfortable reporting their negative experiences with a research team that they perceived as working for the same healthcare facility where they faced disrespect and abuse. This review only considered facility-based abortion care experiences, which necessarily exclude disrespect and abuse experienced by pregnant women who seek out abortion care from pharmacists, traditional healers, or other untrained or illegal providers, which should be explored in future research.

While all studies but one used the terms “women” or “girls” to describe the people who experience abortion care or PAC, female-to-male transgender men may also experience pregnancy and access abortion and PAC (145). One qualitative study in this review included two participants who identified as transgender or gender non-binary (73). The exclusion of the experiences of transgender men and other gender identities is both a limitation of this review and a recommendation for future research.

Disrespect and abuse in abortion and PAC need to be addressed through changing attitudes, policies, and laws to ensure safe access to quality abortion and PAC (36). HCPs need to be supported through education and institutional policies that ensure the provision of kind and compassionate, quality abortion and PAC. Providers should be sensitised to trust women in their decision-making and to understand their emotional needs, creating support groups for women and developing mental health resources that address women's emotional and psychosocial needs. Disrespectful, abusive, and stigmatising behaviours and attitudes experienced by women during their abortion care and PAC can affect women's long-term emotional and psychological health and well-being (146). In contrast, women appreciated HCPs who respectfully talked to them about the emotional and psychosocial impacts of abortion, highlighting the positive role that HCPs can play in supporting women (72, 102).

## 5 Conclusions

This systematic review offers a comprehensive summary of disrespect and abuse in facility-based abortion care and PAC, drawing from 38 studies across 20 countries. This review underscores the multifaceted nature of disrespect and abuse in abortion care and PAC services, ranging from inadequate information to physical violence as well as the presence of abortion protestors. The findings highlight the need for a systemic approach to documenting these issues and implementing multilevel strategies to improve HCPs' perceptions and the quality of abortion care and PAC. Addressing the disrespect and abuse encountered by women in this review requires a nuanced understanding of the complex interplay between individual, societal, institutional, and legal factors that contribute to these negative experiences. Future research should focus on developing quantitative measures for disrespect and abuse in abortion care and PAC, understanding the experiences of vulnerable and marginalised populations, and advocating for policy changes to ensure comprehensive access to SRH services for all, particularly adolescents, transgender men, and other marginalised groups. Safe, respectful, and high-quality abortion care and PAC is a central part of promoting and protecting individuals' SRH and rights, reducing maternal mortality, and achieving gender equity.

## Data Availability

The original contributions presented in the study are included in the article/Supplementary Material, further inquiries can be directed to the corresponding author.
