# Supplementary material for: “One's life becomes even more miserable when we hear all those hurtful words”. A mixed methods systematic review of disrespect and abuse in abortion care
Source: Front Reprod Health. 2025 May 15;7:1561707. doi: 10.3389/frph.2025.1561707 (PMC12119581; doi:10.3389/frph.2025.1561707)
Supplement: Supplementary file 1 [file Table1.docx]

**Appendix**

**Table S1. Embase & Medline Search Strategies**

Searches conducted on: February 19, 2019 and January 19, 2023

|  | **#** | **EMBASE & Medline search strategies** | **Results** |
| --- | --- | --- | --- |
| **ABORTION** | 1 | ‘unsafe abortion’:ti,ab OR ‘unsafe abortions’:ti,ab OR ‘induced abortion’:ti,ab OR ‘induced abortions’:ti,ab OR ‘legal abortion’:ti,ab OR ‘legal abortions’:ti,ab OR ‘abortion’:ti,ab OR ‘abortions’:ti,ab OR ‘spontaneous abortion’:ti,ab OR ‘spontaneous abortions’:ti,ab | 2019: 65,415  2023: 11,895 |
| **DISRESPECT & ABUSE** | 2 | ‘disrespect’:ti,ab OR ‘disrespects’:ti,ab OR ‘disrespectful’:ti,ab OR ‘disrespected’:ti,ab OR ‘respectful’:ti,ab OR ‘abuse’:ti,ab OR ‘abused’:ti,ab OR ‘abusive’:ti,ab OR ‘abuses’:ti,ab OR ‘neglect’:ti,ab OR ‘neglected’:ti,ab OR ‘neglects’:ti,ab OR ‘confidentiality’:ti,ab OR ‘confidential’:ti,ab OR ‘non-confidential’:ti,ab OR ‘informed consent’:ti,ab OR ‘violence’:ti,ab OR ‘violent’:ti,ab OR ‘humiliation’:ti,ab OR ‘humiliate’:ti,ab OR ‘condescend’:ti,ab OR ‘condescending’:ti,ab OR ‘condescension’:ti,ab OR ‘intimidation’:ti,ab OR ‘intimidate’:ti,ab OR ‘yelling’:ti,ab OR ‘yell’:ti,ab OR ‘non dignified’:ti,ab OR ‘non-dignified’:ti,ab OR ‘undignified’:ti,ab OR ‘discrimination’:ti,ab OR ‘discriminate’:ti,ab OR ‘abandon’:ti,ab OR ‘abandonment’:ti,ab OR ‘detention’:ti,ab OR ‘human rights’:ti,ab OR ‘maltreatment’:ti,ab OR ‘mistreatment’:ti,ab OR ‘humanization’:ti,ab OR ‘humanized’:ti,ab OR ‘dehumanized’:ti,ab OR ‘dehumanization’:ti,ab OR ‘dignified’:ti,ab OR ‘undignified’:ti,ab OR ‘stigma’:ti,ab OR ‘dignity’:ti,ab OR ‘bullying’:ti,ab OR ‘bully’:ti,ab OR ‘protest’:ti,ab OR ‘protests’:ti,ab OR ‘protested’:ti,ab OR ‘protesting’:ti,ab OR ‘protestation’:ti,ab OR ‘protestations’:ti,ab | 2019: 600,713  2023: 200,574 |
|  | 3 | #1 AND #2 | 2019: 2,542  2023: 838 |

**Table S2. PubMed Search Strategy**

Search conducted on February 19, 2019 and January 19, 2023

|  | **#** | **Searches** | **Results** |
| --- | --- | --- | --- |
| **ABORTION** | 1 | “abortion” [tiab] OR “abortions” [tiab] OR “unsafe abortion” [tiab] OR “unsafe abortions” [tiab] OR “abortion, induced” [MeSH] OR “induced abortions” [tiab] OR “legal abortion” [tiab] OR “legal abortions” [tiab] OR "abortion, spontaneous"[MeSH] OR "spontaneous abortion" [tiab] | 2019: 90,517  2023: 11,288 |
| **DISRESPECT AND ABUSE** | 2 | “disrespect”[tw] OR “disrespects”[tw] OR “disrespectful”[tw] OR “disrespected”[tw] OR “respectful”[tw] OR “abuse”[tw] OR “abused”[tw] OR “abusive”[tw] OR “abuses”[tw] OR “neglect”[tw] OR “neglected”[tw] OR “neglects”[tw] OR “confidentiality”[tw] OR “confidential”[tw] OR “non-confidential”[tw] OR “informed consent”[tw] OR “violence”[tw] OR “violent”[tw] OR “humiliation”[tw] OR “humiliate”[tw] OR “condescend”[tw] OR “condescending”[tw] OR “condescension”[tw] OR “intimidation”[tw] OR “intimidate”[tw] OR “yelling”[tw] OR “yell”[tw] OR “non dignified”[tw] OR “non-dignified”[tw] OR “undignified”[tw] OR “discrimination”[tw] OR “discriminate”[tw] OR “abandon”[tw] OR “abandonment”[tw] OR “detention”[tw] OR “human rights”[tw] OR “maltreatment”[tw] OR “mistreatment”[tw] OR “humanization”[tw] OR “humanized”[tw] OR “dehumanized”[tw] OR “dehumanization”[tw] OR “dignified”[tw] OR “undignified”[tw] OR “stigma”[tw] OR “dignity”[tw] OR “bullying”[tw] OR “bully”[tw] OR “protest” [tw] OR “protests” [tw] OR “protested” [tw] OR “protesting” [tw] OR “protestation” [tw] OR “protestations” [tw] | 2019: 613,665  2023: 169,272 |
|  | 3 | #1 AND #2 | 2019: 5,225  2023: 820 |

**Table S3. Methodologies and quality assessment of included qualitative studies, based on** **criteria suggested in a cross-disciplinary expert**

**review of quality criteria for qualitative studies.^39^**

| **Author, Year, Area, Country** | **Methodology and Study Recruitment** | **Data Ascertainment** | **Quality Assurance Methods** | **Analytical Methods** | **Principal Findings** |
| --- | --- | --- | --- | --- | --- |
| Altshuler et al., 2017, Northeastern US^1^ | Qualitative Methods for D & A outcomes; Participants were recruited through Craigslist advertisements, community college and public library postings. An obstetrics/gynecologist-researcher interviewed participants over the phone and in-person in a non-medical setting. | Self-reported abortion; phone or in person interview for D & A outcomes | Interviews professionally transcribed and 2 reviewers developed the codebook and consulted with a 3^rd^ reviewer to develop theoretical perspectives on data. | Dedoose used for qualitative data. Developed codebook and used grounded theory. | Women want to be supported as decision-makers, be able to determine their degree of awareness during abortion, & have care provided discreetly and judgment-free. |
| Baum et al., 2021, Mumbai, India and Eldoret and Thika, Kenya^2^ | Abortion clients were recruited from high-client-flow health facilities at their follow-up visits or at the end of their procedure visit. Interviewers knowledgeable about abortion provision led IDIs in India and Kenya, and FGDs in India in a private space in each clinic. | Self-reported abortion; in-person interviews for D & A outcomes. | Methodology took into account cultural norms and perceived acceptability within the two country contexts. Interview guide piloted, and interviews were transcribed. Thematic analysis with inductive techniques were used to identify patterns of priorities in quality of care among all participants. | Dedoose used to develop the codebook. Wrote summaries of key codes to identify patterns and analyse relevant themes. | Patients identified good client-provider relationship as essential for receiving high-quality care. This includes being treated with kindness and dignity, receiving adequate information, reassurance and support from their provider. |
| Belizan et al., 2020, Tegucigalpa, Honduras^3^ | Semi-structured IDIs and FGDs performed in two public healthcare facilities with women attending PAC. Women were interviewed by female trained data collectors. Data was collected in three rounds. | Self-reported abortion; D & A outcomes ascertained through in person interviews. | Questionnaire and interview guide piloted and adapted to local context. Three investigators inductively identified main concepts from the transcripts. | Transcripts coded in ATLAS.ti using adapted grounded theory and thematic analysis. | Women reported barriers to access healthcare services, poor quality of post-abortion care services, mistreatment and abuse by providers. |
| Bennett, 2001, Lombok, Indonesia^4^ | Study draws on ethnographic fieldwork, including 35 IDIs with single women, four IDIs with older, married women and 8 FGDs with 58 participants all of whom have had an abortion. | Self-reported abortion; D & A outcomes ascertained through in person interviews. | NR | NR | Single women experienced poor quality abortion care due to the social stigma around premarital sex and faced D & A from providers. |
| Bercu et al., 2022, Addis Ababa, Aksum, Mek’ele, Ethiopia^5^ | Women were recruited from both private clinics and public health facilities. Potential participants from private clinics were contacted through a call center, where the coordinator called participants after their abortion to enquire about their interest in participating in the study. Women identified through public health facilities were approached with the help of health facility staff. Semi-structured IDIs were held in a private location and lasted between 40 and 60 minutes. | Abortions identified through clinics; D & A outcomes ascertained through in person interviews. | Interview guides developed based on relevant frameworks and adjusted for Ethiopian participants to ensure clarity and cultural applicability. Quality assurance checks were made to ensure accurate translation of the transcripts to English. Two transcripts were double coded to ensure intercoder reliability. | Coding was conducted with MAXQDA with the help of the developed codebook. Thematic analysis was conducted by grouping together related codes. | Participants reported facing disrespect and judgment at healthcare facilities and experienced providers as gatekeepers to legal abortion services. |
| Brack et al., 2018, Bogota, Colombia^6^ | Women who had an abortion in the last 12 months and showed verbal competence in Spanish were recruited through workers at the health facility clinics. Interviews were conducted face-to-face in Spanish in a private office in the clinic and lasted from 30 minutes to 2 hours. | Medical staff reported abortion patients; D & A outcomes ascertained through in person interviews. | A Colombian transcribed interviews to maintain cultural nuances in data. | MAXQDA used to analyze interview data. A phenomenological approach was used to focus on the experiences of each individual. | Physical, emotional, financial, cultural barriers exist around receiving safe abortion services. The lack of training and conscientious objection affected the care provided. |
| Brandi et al., 2018, US^7^ | The research team approached eligible women after their appointment to schedule interested patients for an interview on another date. Semi-structured IDIs were conducted in a private, non-clinical setting by a female clinical researcher. The Integrated Behavioral Model and Reproductive Autonomy Scale were used to develop the interview guide. | Abortions identified by medical records of women in clinic; D & A outcomes ascertained through in person interviews. | Used a validated scale to establish the interview guide. Piloted interview guides, and 2 researchers coded half of the interviews to ensure high reliability. | NVivo software was used for data analysis. Modified grounded theory used to gather key themes | 42% of women felt pressure to choose contraception and 26% of those felt providers were pressuring them to choose LARC methods, Women given more time to consider contraception reported higher reproductive autonomy. |
| Cárdenas et al. 2018, Uruguay^8^ | Women were recruited at a major public hospital in Montevideo, Uruguay after their second appointment. Women were invited by a nurse to participate in the study and if interested provided their contact information. 10 women were chosen by convenience sampling. After their 4^th^ appointment, interviews were conducted in person. Interviews were conducted by trained study coordinators and lasted 30 to 90 minutes. | Abortion care identified by nurses at the hospital. D & A outcomes ascertained through in person interviews. | All codes in codebook were individually scrutinized and the entire research team developed key themes in response. In-vivo codes were also used. | Dedoose used to code and analyze interview data. Thematic analysis was conducted by two researchers. | Patients expressed satisfaction with implementation of new law in Uruguay but still face several barriers to access abortions. Stigmatizing attitudes persist among healthcare staff. |
| Cano and Foster, 2016, Yukon territory, Canada^9^ | Women who had had an abortion on/ after January 1, 2005 were eligible for recruitment. Recruitment strategy included advertisement on listservs, online platforms, and social media, and through local organizations. Interviews lasted 60 minutes on average. | Self-reported abortions. D & A outcomes ascertained through phone/skype interviews. | Iterative multiphase analytic process centering on content and themes. Codebook developed a priori, then adjusted with inductive analytic techniques. Group meetings and discussions guided the interpretation of the results. | Data were managed with ATLAS.ti. One researcher was the primary coder, another reviewed both the evolving codebook and coded transcripts. | Women reported long wait times and found the process with multiple pre-procedure appointments at multiple locations with multiple health-care providers physically, emotionally, and financially draining. |
| Carroll and White, 2020, Louisiana, US^10^ | Clinic staff at health-care facilities referred patients that were attending an abortion-related visit to an on-site research assistant. Semi structured in-depth interviews were conducted within 3 weeks of recruitment over the phone. Interviews were conducted by two trained research assistants with extensive knowledge of abortion service delivery. Interviews lasted 30 minutes on average. | Identified and referred by medical staff. D & A ascertained through interviews over the phone. | Research team independently coded four transcripts using a preliminary coding scheme and refined the codebook afterwards. | Transcripts were coded independently by two researchers. Discrepancies were resolved through discussion with the senior author. | Participants voiced complaints about too few facilities providing abortion, long waiting times and lack of privacy due to protests and congested waiting rooms. |
| Deitch, 2019, Democratic Republic of Congo^11^ | Participants were identified from PAC registers and approached to participate in the study and 2-3 women per facility were selected who have accessed PAC within the last three months. IDIs were conducted by trained facilitators in the participants’ local languages in a private room at the health facilities. Interviews lasted 25-45 minutes. | Identified through PAC registers; D & A outcomes ascertained through in person interviews. | Researchers reviewed transcripts to check the quality of translation to French. Codebook first defined through an inductive approach, then reviewed, and adjusted. Transcripts were coded by two researchers independently. Selected transcripts were coded by a third researcher to ensure reliability and validity. The consistency of coding was assessed by intercoder reliability. Disagreements discussed and resolved until the interrater agreement was in the 90th percentile range. | Transcripts coded independently by 2 researchers in NVivo. | Women’s experiences of PAC at facilities were mostly positive especially with regards to patient-provider interactions. One woman who presented to the facility after an induced abortion reported being ridiculed, another was left with unanswered questions about her miscarriage. Some women reported inadequate pain management by providers. |
| Dennis et al., 2015, Massachusetts, US^12^ | Participants were recruited through posted flyers on Craigslist and in community-based organizations around Massachusetts. Women were invited to contact the research team if interested, and if eligible a telephone interview was scheduled. Interview guides focused on 1) health insurance 2) paying for an abortion and 3) paying for contraceptives. | Self-reported abortion; phone interviews used to ascertain D & A outcomes. | All coding was reviewed by another reviewer to ensure intercoder reliability. | ATLAS software used to analyze interview data. Data discussed with research team to develop major themes. | Women had access to quality abortion care, however some women said they needed better emotional support and privacy. |
| DePiñeres et al., 2017, Bogota, Colombia^13^ | Interviewers approached women after their medical visits to screen for eligibility and obtain consent. Interviews were conducted right after with women for about 15 minutes. Women were also contacted 2 months after for a longer interview to find out women’s experiences after being denied an abortion. | Self-reported abortion; D & A ascertained through in-person interviews and phone interviews | Coding and transcripts were analyzed repeatedly and the entire team review key themes. | Data analyzed using a qualitative content analysis approach. Data was analyzed using Dedoose and synthesized using Excel. | Women faced barriers to abortion services before being denied care. Many women received poor care and faced stigmatizing experiences when obtaining abortions. |
| Doran and Hornibrook 2016, Rural NSW, Australia^14^ | Women were recruited through flyers on local community boards, in public restrooms, media releases, and women’s services. Women interested contacted the researcher and chose to have a phone or face-to-face interview. The interview guide focus on 1) knowledge on abortion clinics 2) logistics to traveling to the clinic 3) follow up care and 4) how to be better supported when accessing an abortion. | Self-reported abortion; D & A ascertained through in-person verbal and phone interviews. | Both authors used data to gather 5 key themes from data. | Thematic analysis was used to analyze interview data. | Women in rural areas experience many barriers to abortion access. Travel, financial, emotional, and informational barriers existed for many women. |
| Foster et al., 2020, Canada^15^ | Women who have had an abortion within the last 5 years were recruited through clinics, community organizations, communities, and social media and virtual spaces. A trained member of the research team conducted semi-structured interviews over the phone or Skype. Interviews lasted 60 minutes on average. | Self-reported abortion; D & A outcomes ascertained through phone/skype interviews. | Interviewers memoed after each interview to reflect on interviewer-participant interaction and engage with interview content. | Data were managed using ATLAS.ti and analyzed for content and themes using inductive and deductive techniques. | Participants’ encounters with protesters in front of clinics did not make them reconsider their decision, however, participants reported feeling upset, stigmatized, and frustrated. |
| Heller et al., 2016, Inverness, Scotland^16^ | Clinic nurses through convenience sampling recruited women. Interviews were conducted 5 weeks to 6 months after the abortion. All interviews were conducted over the phone using a flexible topic guide that addressed experiences, attitudes, and logistics around their abortion. | Clinic nurses identified and approached women undergoing abortion care; D & A ascertained through phone interviews | Two researchers revised the coding framework after independently reviewing the codes and themes. | Thematic analysis using systematic doing was used to gather broad themes. NVivo was the data analysis software used. | Major barriers to access abortions include traveling, temporal factors, attitudes of healthcare staff, and stigma around abortion in Scotland. |
| Kebede et al., 2018, Addis Ababa, Ethiopia^17^ | Recruitment occurred at 3 referral hospitals and 5 health centers where the nurse or physician treating a patient for an incomplete abortion recruited their patients. Individual, repeat interviews were conducted in Amharic and lasted 2-3 hours. | Medical staff referred women undergoing postabortion care; D & A ascertained through in-person interviews. | Repeat interview design chosen to explore themes and gain full understanding of women’s experiences. Guides for second interviews were developed based on each woman’s first interview. | Reflexive and thematic analysis used to gather themes in data. | Young, unmarried women felt their only choice was unsafe abortion services due to their affordability and their privacy. Social stigma around abortion instilled fear in many women. |
| Kilander et al., 2018, Sweden^18^ | Midwives and gynecologists recruited women during their abortion counseling. Purposive sampling was used and women who were eligible and interested were contacted via email or telephone. Interviews were conducted 4-6 weeks after the abortion and 8 interviews were performed in the women’s home, 4 were in a private location, and 1 was conducted over Skype. Interviews lasted around 50 minutes. | Medical staff who met women during abortion counseling identified abortion patients; D & A ascertained through in-person interviews or skype interviews. | Interviews guides piloted before data collection. Data analysis was performed independently by 3 researchers who discussed and agreed on findings and themes | Interview data analyzed using the Moustaka’s modification of the Stevick-Colaizzi-Keen method to identify themes. | Women need more respectful contraceptive counseling during post-abortion care and need guidance when choosing contraception. |
| LaRoche et al., 2021, Australia^19^ | Twenty women and two transgender or gender non-binary people took part in the study, all of whom reported to have had at least one abortion using the drug mifepristone obtained through a variety of health service delivery settings. Recruitment process included liaising with community groups and organizations, social media, and online advertising. Interviews were conducted by the researcher through phone call or Skype with the help of an interview guide containing mostly open-ended questions. | Self-reported abortions; D & A ascertained through interviews via phone call or Skype. | Interview guide modified after second interview to adhere to newly emerging topics. Interviewer memoed after each interview. Initial codebook created inductively and then redefined. Disagreements resolved through discussion. | Content and thematic analyses using deductive and inductive techniques. Data were managed in ATLAS.ti. | Women reported fearing denied care due to the criminalization of abortion and being confused about the legal status of abortion. |
| MacFarlane et al., 2017, Istanbul, Turkey^20^ | A multimodal recruitment strategy including social media posts and outreach via reproductive health organizations was used. Interview guides focused on demographics, reproductive health history, and process of getting abortion care, and how services could be improved. | Self-reported abortions; D&A ascertained through in person interviews. | Main interviewer memoed notes during and after interview to critically reflect on interview dynamics and identify themes. | Content and thematic analysis used to identify key themes. ATLAS software used to code and manage data. | Women receiving abortions in a private facility had more positive experiences. Unmarried women reported more challenges to abortion access. |
| Madeiro and Rufino, 2017, Teresina, Brazil^21^ | Women admitted to a public referral hospital in Teresina were included if eligible and consented to the study. | Medical records were reviewed to identify abortions; D & A outcomes ascertained through in person verbal interviews. | Independent reviewers to code and review data/ | Interviews were read and coded by 2 independent reviewers who compared patterns and determined key themes. | A third of the women interviewed experienced maltreatment and discrimination. |
| Margo et al., 2016, South Carolina, US^22^ | Women were recruited through convenience sampling amongst those who came in for abortions during the days researchers were on-site. The interview guide focused on knowledge around abortion services, experiences with healthcare staff, and barriers to accessing services. Interviews were conducted in a private room at the clinic and lasted 10-30 minutes. | Self-reported receiving abortion care; D & A ascertained through in person interviews. | Independent review of interviewers to ensure reliability. Coding conflicts resolved through discussion. | Thematic analysis approached used. NVivo used to code and compare intercoder reliability. Process mapping used to construct women’s paths to accessing abortion care. | Financial difficulties were the largest barrier to access abortion care along with transportation barriers. Women commonly experienced stress & stigma with their decision to get an abortion. |
| McCallum et al., 2014, Salvador, Brazil^23^ | Women were contacted post-abortion but while they were still in the hospital. Interviews were conducted after they left the hospital in locations that the participants chose. | Medical staff identified women undergoing abortion care; D & A outcomes through in person interviews | NR | NR | Discrimination against women who have had abortions in deeply embedded within the health institution’s structure and culture and not solely due to the individual healthcare provider. |
| Mutua et al., 2018, Kenya^24^ | Purposive sampling was used to include only health facilities that dealt with a high volume of PAC. A sample of 6 out of the 16 facilities was included where 5 patients were recruited from each facility. However, only 21 patients were interviewed. Patient interviews lasted on average 21 minutes. Women who were treated for PAC were interviewed after their appointment. | Women identified as accessing PAC through medical records were interviewed; D & A outcomes ascertained through in person interviews. | Reviewers listened to audio-recordings of interviews to determine quality and ensure improvement by providing feedback to interviewers. | Data analyzed deductively and inductively between two reviewers. NVivo was used to manage data. | Poorly trained providers, negative attitudes towards abortion, a lack of resources, and a lack of capacity within facilities hinder postabortion care services. |
| Netshinombelo et al., 2022, KwaZulu-Natal, South Africa^25^ | The study was conducted at all 23 hospitals in the province that provided post abortion care and 23 women, one from each hospital, who presented with incomplete abortion and accessed PACs were recruited and purposely sampled. Unstructured face-to-face IDIs were conducted in a quiet private room at the health care facility. Interviews lasted between 30-45 minutes. | Women contacted at health care facilities (not further described). D & A outcomes ascertained through in-person interviews. | Recorded interviews were translated word by word, including nonverbal cues, to English by a language expert. Researchers used a qualitative method to mitigate potential bias and potential deleterious effects of unacknowledged preconceptions related to the research. | A qualitative explorative, descriptive, and contextual approach was used. | The main challenges reported by women when accessing PAC included transportation barriers, stigma, and mistreatment. |
| Otsin et al., 2022, Ashanti, Ghana^26^ | Women seeking PAC at health care facilities after unsafe abortions were recruited through advertising materials placed at hospitals. Additionally, healthcare workers informed patients who fit the inclusion criteria about the study. Semi-structured IDIs were conducted by the lead researcher in person in the local language. Interviews lasted between 30 and 75 minutes. | Self-reported PAC; D & A ascertained through in person interviews. | NR | Drew on phenomenology for detailed analysis of participants’ experiences. Inductive coding. | Three delays identified for women’s access to appropriate abortion care: 1) women’s poor knowledge of pregnancy and influence of religion; 2) cost, provider attitudes, stigma, and proximity of pharmacies; 3) hospitals’ non-prioritization of abortion complications and shortage of equipment resulting in longer waiting times. |
| Ouedraogo and Juma, 2020, Ouagadougou, Burkina Faso^27^ | Data were gathered through IDIs. Recruited women who have had abortions from health facilities. Interviews were designed to simulate an informal discussion with the help of a pre-tested semi-structured interview guide. Interviews took place in various places depending on participants’ preferences. | Identified from healthcare facilities; D & A ascertained through informal interview. | NR | Data were analyzed using a thematic analysis approach. | Findings describe a devalorized image of PAC driven by abortion stigma. Some reported fearing potential violence from healthcare providers because of not approving their decision for abortion, and therefore opted for medication abortion. |
| Penfold et al., 2018, Western Kenya^28^ | Women who were interviewed on the day of the procedure for the pre-intervention phase of the study were invited for a second interview 3 months later. Interested respondents were systematically sampled and interviews were conducted face-to-face in a private location and lasted up to 1 hour. Interview guides focused on abortion-seeking behavior, knowledge of abortion providers, and experiences obtaining abortion. | Self-reported abortion or PAC; D & A ascertained through in-person interviews. | 2 coders reviewed the first 4 interviews to reach consensus on coding structure. | Descriptive thematic analysis using inductive and deductive coding was done. | Women reported wanting to use abortion services at clean, safe center with respectful, private providers. Many women experienced complex pathways to seeking safe abortion care. |
| Puri et al., 2015, Nepal^29^ | Clinic staff recruited women the day they were denied an abortion and were contacted 2 months after recruitment for an interview. Participants were a purposive sample focused on diversity. Health facility providers noted the reason women were denied services. IDIs were conducted face-to-face in Nepali and translated to English. The interview guide was open-ended and focused on abortion decision-making processes, experiences with abortion denial, future plans, and advice for others wanting an abortion. | Women denied abortion services were approached by research team; D & A outcomes ascertained through in-person interviews | 2 independent reviewers trained in qualitative analysis coded the data to ensure reliability of codes and clarity of codebook. | Thematic analysis was used to identify key themes in data. Dedoose was the analytical software used to manage data. | Women most commonly cited financial and health reasons for seeking abortions. After women were denied abortion care, 12 decided to continue their pregnancy, 12 terminated elsewhere, and 1 self-induced using medication abortion. |
| Sunil, 2022, Tamil Nadu, India^30^ | Participants were selected using a purposive, referral-based, and respondent-driven strategy. Eligible women were married and had experienced at least one induced abortion for reasons other than fetal anomaly and sex selection. Snowball sampling was used to identify participants. IDIs were conducted using an interview guide across multiple sessions suiting the respondent’s availability. Interviews lasted on average about two hours. | Snowball sampling through healthcare workers; D & A outcomes ascertained through in person interviews. | NR | Phenomenological approach to data analysis. Transcripts thematically coded using ATLAS.ti. | Women face many challenges when seeking abortion including receiving misleading information from frontline workers, and judgment, disrespect, and delaying tactics from providers. |

D & A: Disrespect and abuse; IDIs: In-depth interviews; FGDs: Focus group discussions; LARC: Long-acting reversible contraception; NR: Not reported; PAC: Postabortion care.

**Table S4. Quality assessment of included quantitative studies, based on the Mixed Methods Appraisal Tool (MMAT) version 2018.^40^**

|  | **Is the sampling strategy relevant to address the research question?** | **Is the sample representative of the target population?** | **Are the measurements appropriate?** | **Is the risk of nonresponse bias low?** | **Is the statistical analysis appropriate to answer the research question?** |
| --- | --- | --- | --- | --- | --- |
| Aguilar et al., 2023, Northeastern US^31^ | Yes | Yes | Yes | Yes | Yes |
| Arey, 2023, NorthCarolina, US^32^ | Yes | Yes | Yes | No | Yes |
| Becker et al., 2011, Mexico City, Mexico^33^ | Yes | Yes | Yes | No | Yes |
| Clyde et al., 2013, Mexico City, Mexico^34^ | Yes | Yes | Yes | No | Can’t tell |
| Makleff et al, 2019, Montevideo, Uruguay^35^ | Yes | Yes | Yes | Yes | Yes |
| Regmi and Madison, 2010, Kathmandu, Nepal^36^ | Yes | Yes | Yes | Yes | Yes |
| Sudhinaraset et al., 2019, Nairobi County,  Kenya^37^ | Yes | Yes | Yes | Yes | Yes |
| Wallin Lundell etal., 2015, Sweden^38^ | Yes | Yes | Yes | No | Yes |

**References**

1. Altshuler AL, Ojanen-Goldsmith A, Blumenthal PD, Freedman LR. A good abortion experience: A qualitative exploration of women's needs and preferences in clinical care. Soc Sci Med. 2017;191:109-16.

2. Baum SE, Wilkins R, Wachira M, Gupta D, Dupte S, Ngugi P, Makleff S. Abortion quality of care from the client perspective: a qualitative study in India and Kenya. Health Policy Plan. 2021;36(9):1362-70.

3. Belizan M, Maradiaga E, Roberti J, Casco-Aguilar M, Ortez AF, Avila-Flores JC, et al. Contraception and post abortion services: qualitative analysis of users' perspectives and experiences following Zika epidemic in Honduras. BMC Womens Health. 2020;20(1):199.

4. Bennett LR. Single women's experiences of premarital pregnancy and induced abortion in Lombok, Eastern Indonesia. Reprod Health Matters. 2001;9(17):37-43.

5. Bercu C, Jacobson LE, Gebrehanna E, Ramirez AM, Katz AJ, Filippa S, Baum SE. “I was afraid they will be judging me and even deny me the service”: Experiences of denial and dissuasion during abortion care in Ethiopia. Frontiers in Global Women's Health. 2022;3.

6. Brack CE, Rochat RW, Bernal OA. "It's a Race Against the Clock": A Qualitative Analysis of Barriers to Legal Abortion in Bogotá, Colombia. Int Perspect Sex Reprod Health. 2017;43(4):173-82.

7. Brandi K, Woodhams E, White KO, Mehta PK. An exploration of perceived contraceptive coercion at the time of abortion. Contraception. 2018;97(4):329-34.

8. Cárdenas R, Labandera A, Baum SE, Chiribao F, Leus I, Avondet S, Friedman J. “It’s something that marks you”: Abortion stigma after decriminalization in Uruguay. Reproductive Health. 2018;15(1):150.

9. Cano JK, Foster AM. "They made me go through like weeks of appointments and everything": Documenting women's experiences seeking abortion care in Yukon territory, Canada. Contraception. 2016;94(5):489-95.

10. Carroll E, White K. Abortion patients' preferences for care and experiences accessing services in Louisiana. Contracept X. 2020;2:100016.

11. Deitch J, Amisi JP, Martinez S, Meyers J, Muselemu JB, Nzau JJ, et al. "They Love Their Patients": Client Perceptions of Quality of Postabortion Care in North and South Kivu, the Democratic Republic of the Congo. Glob Health Sci Pract. 2019;7(Suppl 2):S285-s98.

12. Dennis A, Manski R, Blanchard K. A Qualitative Exploration of Low-Income Women's Experiences Accessing Abortion in Massachusetts. Womens Health Issues. 2015;25(5):463-9.

13. DePiñeres T, Raifman S, Mora M, Villarreal C, Foster DG, Gerdts C. ‘I felt the world crash down on me’: Women’s experiences being denied legal abortion in Colombia. Reproductive Health. 2017;14(1):133.

14. Doran FM, Hornibrook J. Barriers around access to abortion experienced by rural women in New South Wales, Australia. Rural Remote Health. 2016;16(1):3538.

15. Foster AM, Persaud MS, LaRoche KJ. “I didn’t doubt my choice, but I felt bad”: A qualitative exploration of Canadian abortion patients’ experiences with protesters. Contraception. 2020;102(5):308-13.

16. Heller R, Purcell C, Mackay L, Caird L, Cameron ST. Barriers to accessing termination of pregnancy in a remote and rural setting: a qualitative study. Bjog. 2016;123(10):1684-91.

17. Kebede MT, Middelthon AL, Hilden PK. Negotiating the social and medical dangers of abortion in Addis Ababa: An exploration of young, unmarried women's abortion-seeking journeys. Health Care Women Int. 2018;39(2):186-207.

18. Kilander H, Berterö C, Thor J, Brynhildsen J, Alehagen S. Women's experiences of contraceptive counselling in the context of an abortion - An interview study. Sex Reprod Healthc. 2018;17:103-7.

19. LaRoche KJ, Wynn LL, Foster AM. "We have to make sure you meet certain criteria": exploring patient experiences of the criminalisation of abortion in Australia. Public Health Res Pract. 2021;31(3).

20. MacFarlane KA, O'Neil ML, Tekdemir D, Foster AM. "It was as if society didn't want a woman to get an abortion": a qualitative study in Istanbul, Turkey. Contraception. 2017;95(2):154-60.

21. Madeiro AP, Rufino AC. [Maltreatment and discrimination in induced abortion care: perception of women in Teresina, State of Piauí, Brazil]. Cien Saude Colet. 2017;22(8):2771-80.

22. Margo J, McCloskey L, Gupte G, Zurek M, Bhakta S, Feinberg E. Women's Pathways to Abortion Care in South Carolina: A Qualitative Study of Obstacles and Supports. Perspect Sex Reprod Health. 2016;48(4):199-207.

23. McCallum C, Menezes G, Reis AP. The dilemma of a practice: experiences of abortion in a public maternity hospital in the city of Salvador, Bahia. Hist Cienc Saude Manguinhos. 2016;23(1):37-56.

24. Mutua MM, Manderson L, Musenge E, Achia TNO. Policy, law and post-abortion care services in Kenya. PLoS One. 2018;13(9):e0204240.

25. Netshinombelo M, Maputle MS, Ramathuba DU. Women's Perceived Barriers to Accessing Post-Abortion Care Services in Selected Districts in KwaZulu Natal Province, South Africa: A Qualitative Study. Ann Glob Health. 2022;88(1):75.

26. Otsin MNA, Taft AJ, Hooker L, Black K. Three Delays Model applied to prevention of unsafe abortion in Ghana: a qualitative study. BMJ Sex Reprod Health. 2022;48(e1):e75-e80.

27. Ouedraogo R, Juma K. From the shadows to light. Perceptions of women and healthcare providers of post-abortion care in Burkina Faso. Soc Sci Med. 2020;260:113154.

28. Penfold S, Wendot S, Nafula I, Footman K. A qualitative study of safe abortion and post-abortion family planning service experiences of women attending private facilities in Kenya. Reprod Health. 2018;15(1):70.

29. Puri MC, Raifman S, Daniel S, Karki S, Maharjan DC, Ahlbach C, et al. Denial of legal abortion in Nepal. PLoS One. 2023;18(3):e0282886.

30. Sunil B. Running an obstacle-course: a qualitative study of women's experiences with abortion-seeking in Tamil Nadu, India. Sex Reprod Health Matters. 2021;29(2):e1966218.

31. Aguilar GA, Lundsberg LS, Stanwood NL, Gariepy AM. Exploratory study of race- or ethnicity-based discrimination among patients receiving procedural abortion care. Contraception. 2023;120:109949.

32. Arey W. Experiences with small and large numbers of protesters at abortion clinics in North Carolina. Contraception. 2023;120:109919.

33. Becker D, Diaz-Olavarrieta C, Juarez C, Garcia SG, Sanhueza P, Harper CC. Clients' perceptions of the quality of care in Mexico city's public-sector legal abortion program. Int Perspect Sex Reprod Health. 2011;37(4):191-201.

34. Clyde J, Bain J, Castagnaro K, Rueda M, Tatum C, Watson K. Evolving capacity and decision-making in practice: adolescents' access to legal abortion services in Mexico City. Reprod Health Matters. 2013;21(41):167-75.

35. Makleff S, Labandera A, Chiribao F, Friedman J, Cardenas R, Sa E, Baum SE. Experience obtaining legal abortion in Uruguay: knowledge, attitudes, and stigma among abortion clients. BMC Womens Health. 2019;19(1):155.

36. Regmi K, Madison J. Ensuring patient satisfaction with second-trimester abortion in resource-poor settings. Int J Gynaecol Obstet. 2010;108(1):44-7.

37. Sudhinaraset M, Landrian A, Montagu D, Mugwanga Z. Is there a difference in women's experiences of care with medication vs. manual vacuum aspiration abortions? Determinants of person-centered care for abortion services. PLoS One. 2019;14(11):e0225333.

38. Wallin Lundell I, Öhman SG, Sundström Poromaa I, Högberg U, Sydsjö G, Skoog Svanberg A. How women perceive abortion care: A study focusing on healthy women and those with mental and posttraumatic stress. Eur J Contracept Reprod Health Care. 2015;20(3):211-22.

39. Santiago-Delefosse M, Gavin A, Bruchez C, Roux P, Stephen SL. Quality of

qualitative research in the health sciences: analysis of the common criteria present

in 58 assessment guidelines by expert users. Soc Sci Med 1982. (2016) 148:142–51.

doi: 10.1016/j.socscimed.2015.11.007.

40. Hong QN, Fàbregues S, Bartlett G, Boardman F, Cargo M, Dagenais P, Gagnon MP, Griffiths F, Nicolau B, O’Cathain A, Rousseau MC. The Mixed Methods Appraisal Tool (MMAT) version 2018 for information professionals and researchers. Education for information. 2018 Nov;34(4):285-91.
